# Fatty acid binding protein 4 promotes autoimmune diabetes by recruitment and activation of pancreatic islet macrophages

**DOI:** 10.1172/jci.insight.141814

**Published:** 2021-04-08

**Authors:** Yang Xiao, Lingling Shu, Xiaoping Wu, Yang Liu, Lai Yee Cheong, Boya Liao, Xiaoyu Xiao, Ruby L.C. Hoo, Zhiguang Zhou, Aimin Xu

**Affiliations:** 1 National Clinical Research Center for Metabolic Diseases, Department of Metabolism and Endocrinology, The Second Xiangya Hospital, Central South University, Changsha, China.; 2 State Key Laboratory of Oncology in South China, Collaborative Innovation Center for Cancer Medicine, Department of Hematologic Oncology, Cancer Center, Sun Yat-Sen University, Guangzhou, China.; 3 State Key Laboratory of Pharmaceutical Biotechnology,; 4 Department of Medicine, and; 5 Department of Pharmacology & Pharmacy, The University of Hong Kong, Hong Kong, China.; 6 Department of Neurosurgery, Zhujiang Hospital, Southern Medical University, Guangzhou, China.

**Keywords:** Autoimmunity, Endocrinology, Diabetes, Innate immunity, Macrophages

## Abstract

Both innate and adaptive immune cells are critical players in autoimmune destruction of insulin-producing β cells in type 1 diabetes. However, the early pathogenic events triggering the recruitment and activation of innate immune cells in islets remain obscure. Here we show that circulating fatty acid binding protein 4 (FABP4) level was significantly elevated in patients with type 1 diabetes and their first-degree relatives and positively correlated with the titers of several islet autoantibodies. In nonobese diabetic (NOD) mice, increased FABP4 expression in islet macrophages started from the neonatal period, well before the occurrence of overt diabetes. Furthermore, the spontaneous development of autoimmune diabetes in NOD mice was markedly reduced by pharmacological inhibition or genetic ablation of FABP4 or adoptive transfer of FABP4-deficient bone marrow cells. Mechanistically, FABP4 activated innate immune responses in islets by enhancing the infiltration and polarization of macrophages to proinflammatory M1 subtype, thus creating an inflammatory milieu required for activation of diabetogenic CD8^+^ T cells and shift of CD4^+^ helper T cells toward Th1 subtypes. These findings demonstrate FABP4 as a possible early mediator for β cell autoimmunity by facilitating crosstalk between innate and adaptive immune cells, suggesting that pharmacological inhibition of FABP4 may represent a promising therapeutic strategy for autoimmune diabetes.

## Graphical abstract

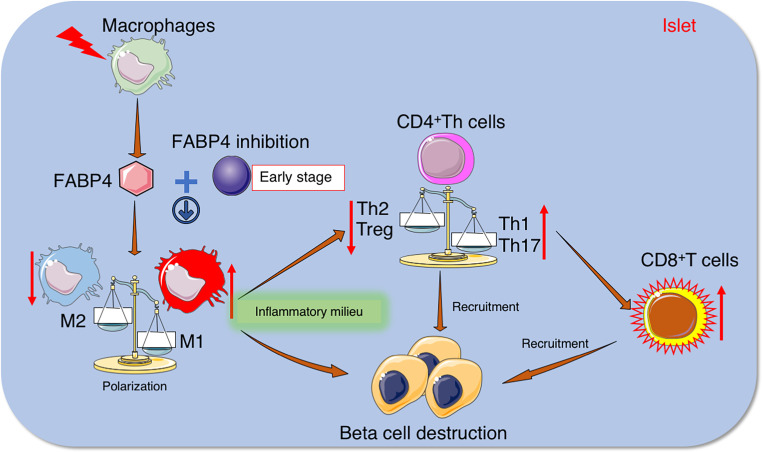

## Introduction

Type 1 diabetes is an organ-specific autoimmune disease caused by the selective destruction of insulin-producing β cells located in pancreatic islets of Langerhans (1). Although the incidence of type 1 diabetes is much lower than type 2 diabetes, the former often occurs at a much younger age and is the most severe type of diabetes. Intensive research in the past several decades has led to the identification of many genetic, immunological, and environmental factors involved in the pathogenesis of type 1 diabetes. However, there is currently no cure available for type 1 diabetes, and most patients have to rely on lifelong insulin injection.

Similar to other autoimmune disorders, type 1 diabetes is a chronic inflammatory disease involving dysfunction of both innate and adaptive immunity. Infiltration and activation of several types of innate immune cells in the islets, including macrophages, neutrophils, natural killer (NK) cells, and dendritic cells (DCs), occur at the initial stage of type 1 diabetes and are the major contributor to the establishment of inflammatory milieu in the islets, which is prerequisite for subsequent β cell death and augmentation of the adaptive immune effector response of islet-specific autoreactive CD4^+^ T cells and cytotoxic CD8^+^ T cells (2, 3). Autoantigens released from β cell damage are shed and phagocytosed by antigen-presenting cells (APCs) including DCs and macrophages, which further exacerbate β cell destruction by releasing proinflammatory factors (such as TNF-α, IL-12, IL-1β, and reactive oxygen species) (4). Macrophages are the major population of infiltrated immunocytes at the early stage of insulitis in NOD mice and diabetes-prone BB rats (5, 6) and are also found in the islets of patients with recent-onset type 1 diabetic patients (7). The transcriptional profiles of macrophages that reside in the islets of 3-week-old NOD mice exhibit an activation signature with high expression of MHC-II, TNF-α, and IL-1β, as well as elevated levels of chemokines and chemokine receptors, suggesting that islet macrophages are poised to mount an immune response even at the time of weaning (8). Moreover, depletion of macrophages with silica or clodronate-loaded liposomes prevents the development of insulitis and diabetes in NOD mice (6, 9). However, the etiologic factors triggering the recruitment and activation of islet macrophages in the early pathogenesis of type 1 diabetes remain obscure.

Fatty acid binding protein 4 (FABP4, also known as A-FABP or aP2) is a member of the FABP family abundantly expressed in adipocytes (10), macrophages (11), and endothelial cells (12). FABP4 functions as a lipid-binding chaperone that regulates trafficking and cellular signaling of fatty acids and plays an important role in linking lipid metabolism with innate immunity and inflammation (13). Circulating FABP4 level is elevated in obese individuals and is an independent predictor for obesity-related cardiometabolic syndrome (14–16). Pharmacological inhibition or genetic ablation of FABP4 has been shown to be effective in alleviating insulin resistance, atherosclerosis, and nonalcoholic fatty liver disease (17, 18). Additionally, FABP4 has been implicated in the pathogenesis of several autoimmune disorders, including asthma and experimental autoimmune encephalomyelitis/multiple sclerosis (19–21). FABP4-deficient mice exhibit a lower incidence of autoimmune encephalomyelitis, reduced clinical symptoms, and dramatically lower levels of proinflammatory cytokines in the brain as compared with WT mice (20). Furthermore, serum FABP4 concentrations were significantly increased and independently associated with poor glycemic control in Chinese children with type 1 diabetes (22) and also predict pre-eclampsia in women with type 1 diabetes (23). However, whether FABP4 contributes to the pathogenesis of autoimmune diabetes has not yet been addressed.

In the present study, we measured the dynamic changes of circulating FABP4 in patients with type 1 diabetes and their first-degree relatives (FDRs) and interrogated its relationship with autoantibody positivity and β cell autoimmunity in these subjects. Furthermore, we investigated the roles of FABP4 in the pathogenesis of insulitis and autoimmune diabetes in NOD mice using both pharmacological and genetic approaches. Our results identify augmented FABP4 expression in islet macrophages as an important mediator triggering the onset of β cell autoimmunity by enhancing inflammatory milieu in the islets, suggesting that pharmacological inhibition of FABP4 might be an effective approach for early therapeutic intervention of autoimmune diabetes.

## Results

### Serum FABP4 is elevated in patients with type 1 diabetes and their first-degree relatives and is closely associated with islet autoantibodies.

**Figure 1:**
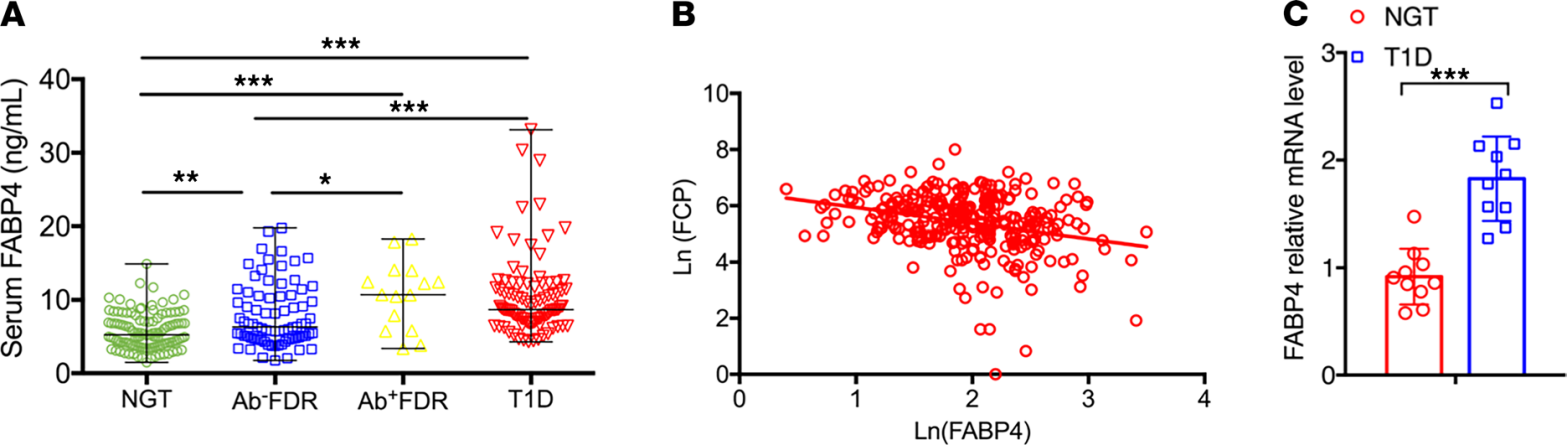
Circulating FABP4 level is elevated in patients with type 1 diabetes and their FDRs and is inversely associated with β cell function. (**A**) The concentration of fasting serum FABP4 in subjects with normal glucose tolerance (NGT, *n* = 102); islet autoantibody–negative FDRs (Ab^−^FDR, *n* = 78); islet autoantibody–positive FDRs (Ab^+^FDR, *n* = 15); and type 1 diabetes patients (T1D, *n* = 92). (**B**) Correlation between serum levels of FABP4 and fasting C-peptide (FCP) in T1D patients. (**C**) The mRNA expression levels of FABP4 in peripheral blood mononuclear cells (PBMCs) isolated from patients and sex- and age-matched individuals with NGT were expressed as arbitrary units after normalization for 16S RNA (*n* = 10). Data are expressed as median ± IQR. Differences between the groups were assessed by χ^2^ test for categorical data or unpaired Student’s *t* test for continuous variables. One-way ANOVA was used for comparisons among groups. Correlations were evaluated with Spearman or partial correlation as appropriate. *P < 0.05, **P < 0.01, ***P < 0.001, after adjustment for age and BMI.

To investigate the association of FABP4 with the development of autoimmune diabetes, we recruited 92 patients with new-onset type 1 diabetes and their FDRs (*n* = 93), including islet autoantibody-negative (Ab^−^FDRs, *n* = 78) and islet autoantibody-positive (Ab^+^FDRs, *n* =15), as well as sex-matched healthy control subjects (*n* = 102) (Table 1). All the Ab^+^FDRs were single autoantibody positive. Circulating FABP4 level in patients with type 1 diabetes was significantly higher than in healthy control subjects (median 8.7 [IQR 7.1–12.1] vs. 5.0 [3.7–6.8] ng/mL; *P* < 0.001). Moreover, circulating FABP4 was obviously increased in Ab^+^FDR subjects (median 10.7 [IQR 5.8–14.0] vs. 5.0 [3.7–6.8] ng/mL; *P* < 0.001), and moderately elevated in Ab^–^FDR individuals (median 6.3 [IQR 4.8–10.6] vs. 5.3 [3.7–7.2] ng/mL; *P* < 0.01), suggesting that augmented FABP4 production occurs well before overt diabetes (Figure 1A). Serum FABP4 concentration in Ab^–^FDR subjects was lower than that of Ab^+^FDR subjects (*P* < 0.05) and type 1 diabetes patients (*P* < 0.001), while there was no difference between the Ab^+^FDR group and type 1 diabetes group (Table 1 and Figure 1A). Correlation analysis showed that serum FABP4 levels were inversely associated with fasting C-peptide (*r* = –0.274, *P* < 0.001, Figure 1B) but were positively associated with the titers of islet autoantibodies GADA (*r* = 0.234, *P* = 0.002) and IA-2A (*r* = 0.234, *P* = 0.002) as well as the quantity of islet autoantibodies (*r* = 0.219, *P* = 0.04) in patients with type 1 diabetes (Table 2). Furthermore, these associations remained significant after adjustment for age and BMI, suggesting that elevated FABP4 may be causally involved in autoimmune destruction of β cells in patients with type 1 diabetes. Consistently, the *FABP4* mRNA expression in peripheral blood mononuclear cells (PBMCs) isolated from patients with type 1 diabetes was significantly higher than that from sex- and age-matched healthy individuals with NGT (Figure 1C), indicating that monocyte-derived FABP4 may contribute to the elevation of serum FABP4 in patients with type 1 diabetes.

### FABP4 is mainly expressed in macrophages of pancreatic islets at the early stage of autoimmune diabetes in NOD mice.

**Figure 2:**
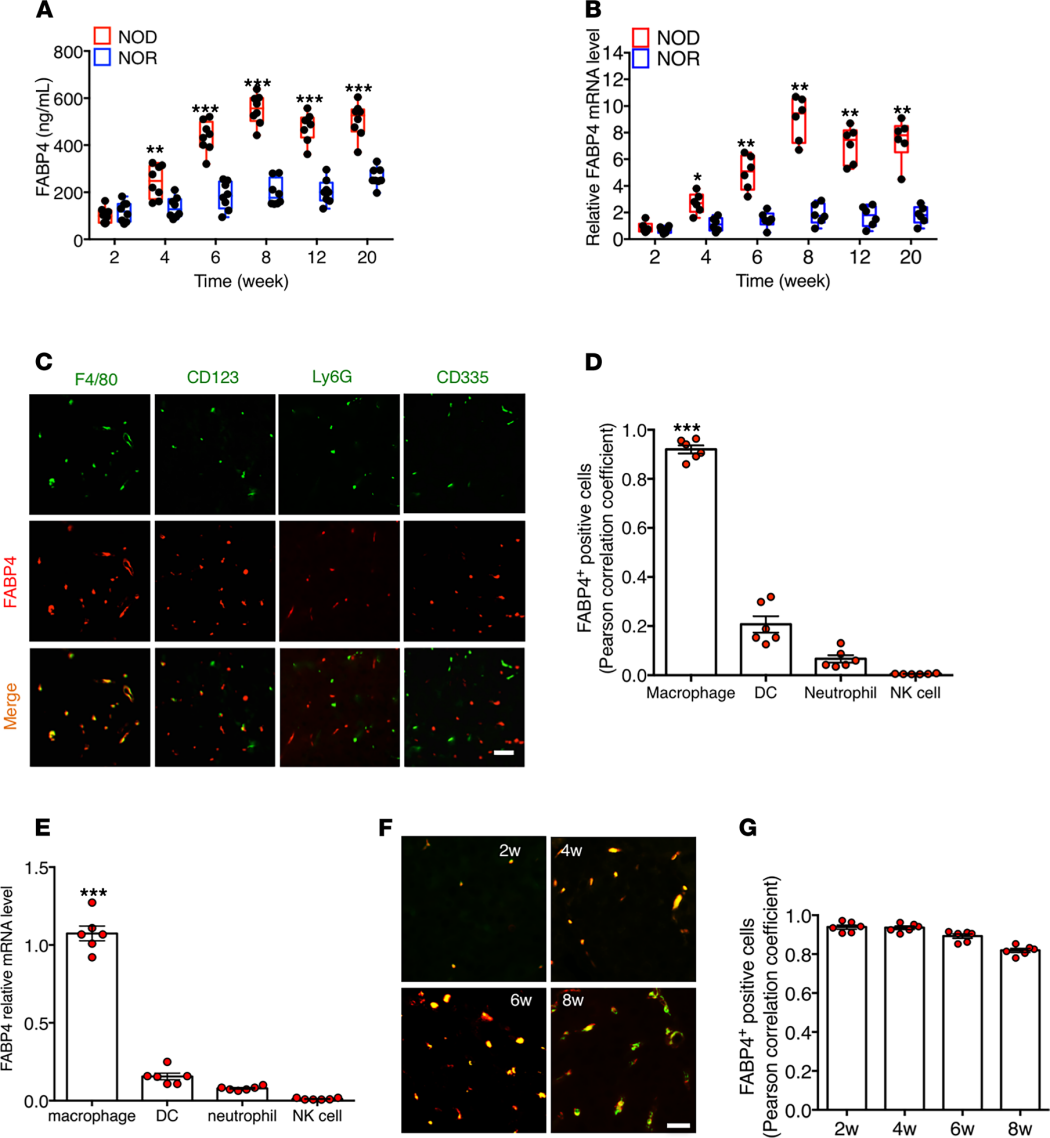
FABP4 expression in pancreatic macrophages is elevated at an early age in NOD mice. (**A**) Dynamic circulating levels of FABP4 in NOD/ShiLtJ and their control NOR/LtJ mice at different ages (*n* = 6). (**B**) The mRNA abundance of *FABP4* in pancreas of NOD/ShiLtJ and NOR/LtJ determined by real-time PCR analysis, expressed as arbitrary units after normalization for *GAPDH* mRNA levels, relative to the levels of 2-week-old NOD mice (*n* = 6). (**C**) Representative images of immunohistochemistry (IHC) staining for FABP4 (red), F4/80 (green), CD123 (green), Ly6G (green), and CD335 (green) in the pancreases of 6-week-old NOD/ShiLtJ mice. Scale bar: 20 μm, with original magnification of 400× (*n* = 6). (**D**) The colocalization of FABP4 with F4/80, CD123, Ly6G, and CD335 was measured by ImageJ (NIH) and expressed as Pearson correlation coefficient (*n* = 6). (**E**) The mRNA abundance of *FABP4* in macrophages, DCs, neutrophils, and NK cells sorted from pancreases of 6-week-old NOD/ShiLtJ NOD mice with flow cytometry (*n* = 6). (**F**) Representative images of immunofluorescence costaining of FABP4 (red) with the macrophage marker F4/80 (green) in islets of NOD/ShiLtJ mice at different ages; scale bar: 20 μm, with original magnification of 400× (*n* = 6). (**G**) The colocalization of FABP4 with F4/80 in (**F**) measured by ImageJ and expressed as Pearson correlation coefficient (*n* = 6). Data are expressed as mean ± standard deviation. Statistical significance was determined by 1-way analysis of variance or Student’s *t* test. *P < 0.05, **P < 0.01, ***P < 0.001.

To explore the pathophysiological roles of FABP4 in autoimmune diabetes, dynamic changes of FABP4 expression were further investigated in circulation and pancreas of NOD/ShiLtJ mice at different ages in comparison with those of NOR/LtJ mice. Circulating FABP4 started to rise at 4 weeks of age, reached a peak level at 8 weeks, and then slightly declined in NOD mice (Figure 2A), and these changes were mirrored by increased pancreatic *FABP4* mRNA expression (Figure 2B). Histologic staining of pancreas from 6-week-old NOD mice showed that FABP4 was mainly colocalized with F4/80^+^ macrophages but had little overlap with CD123^+^ DCs, Ly6G^+^ neutrophils, and CD335^+^ NK cells (Figure 2, C and D). Flow cytometry analysis showed that macrophages are the most abundant innate immune cells infiltrated in islets (Supplemental Figure 1, A and B; supplemental material available online with this article; https://doi.org/10.1172/jci.insight.141814DS1). When these innate immune cells in the pancreas of 6-week-old NOD mice were further sorted with flow cytometry and subjected to quantitative real-time PCR analysis, the result confirmed that macrophages, but not DCs, neutrophils, and NK cells, were the major site for *FABP4* expression (Figure 2E). Further immunocytochemistry analysis for the pancreas from different ages of mice demonstrated that both FABP4 and F4/80^+^ macrophages became detectable from 2 weeks of age and remained almost completely overlapped until 6 weeks of age (Figure 2, F and G). Taken together, these findings suggest that FABP4 is predominantly expressed in macrophages at the early stage of autoimmune diabetes.

### Pharmacological inhibition of FABP4 at an early age delays onset time and reduces the incidence of diabetes in NOD mice.

**Figure 3:**
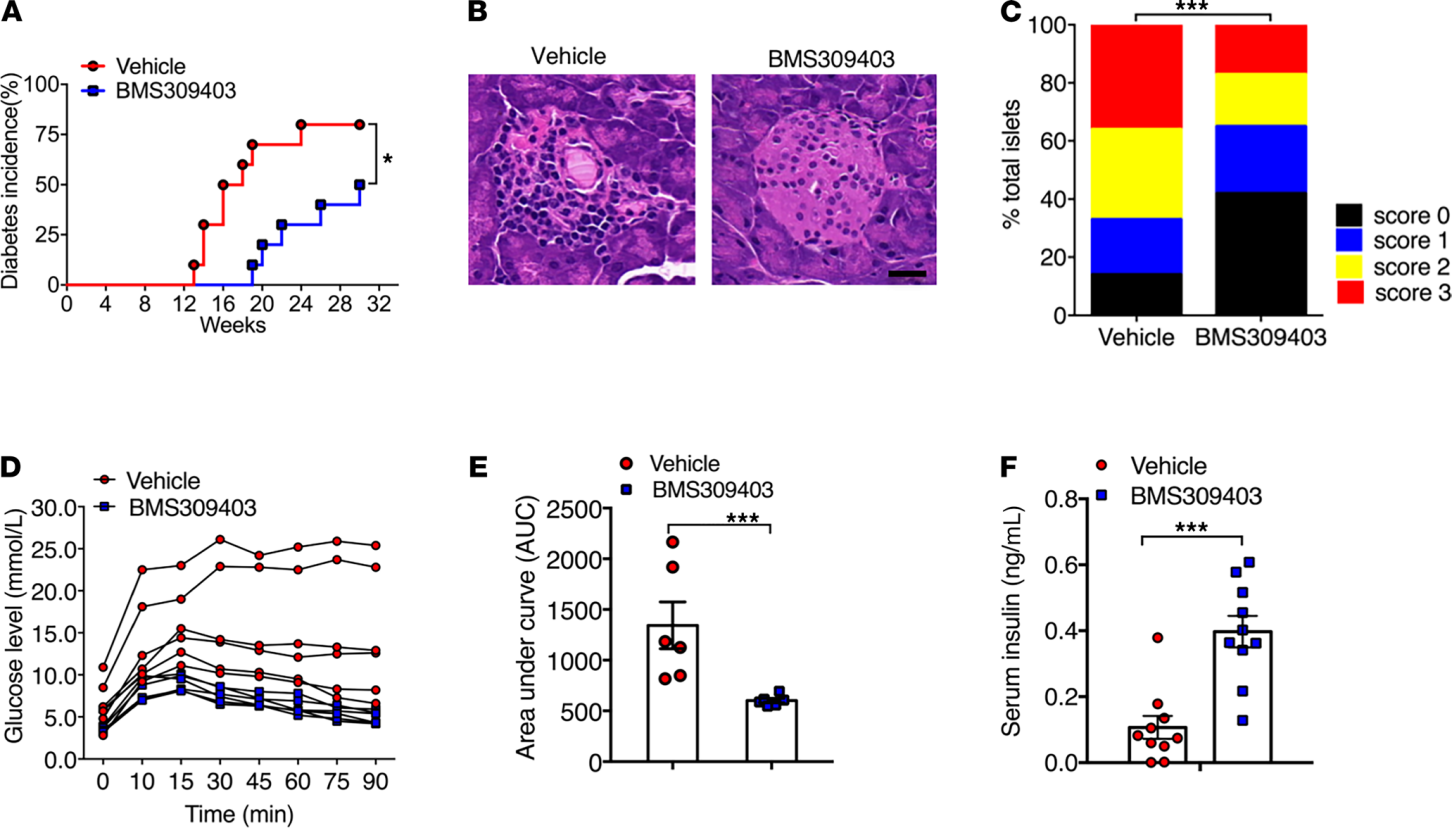
Pharmacological inhibition of FABP4 ameliorates spontaneous development of insulitis and diabetes in NOD mice. Female 4-week-old NOD mice were treated with BMS309403 (40 mg/kg/day) or vehicle (PBS) by oral gavage for 8 weeks, followed by monitoring diabetes incidence once a week until 30 weeks of age. (**A**) Incidence of diabetes expressed as percentage of diabetic mice at different ages. Diabetes incidence was diagrammed with the Kaplan-Meier method, and incidences between different groups were compared with the log-rank test (*n* = 20). (**B**) Representative hematoxylin and eosin (H&E) staining of pancreases from 12-week-old NOD mice treated with BMS309403 or vehicle (*n* = 6). (**C**) Insulitis scores calculated based on histological evaluation of pancreatic section. Score 0 = no insulitis; score 1 = peri-islet insulitis; score 2 = intra-islet insulitis; score 3 = complete islet insulitis. (**D**) Glucose excursion curve of each individual mouse during glucose tolerance test and (**E**) area under curve for glucose tolerance test (*n* = 6). (**F**) Fasting serum insulin concentration in 16-week-old BMS309403 or vehicle-treated NOD mice (*n* = 10). Data are expressed as mean ± standard deviation. Statistical significance was determined by 1-way analysis of variance or Student’s *t* test. *P < 0.05, **P < 0.01, ***P < 0.001.

The pharmacological inhibitors of FABP4 have been shown to be effective in treating various inflammatory diseases, including nonalcoholic steatohepatitis and vascular inflammation in both rodents and pigs (17, 18). Therefore, we evaluated the effect of BMS309403, a cell-permeable, highly potent, and selective inhibitor of FABP4 that targets the fatty acid binding pocket (24), on spontaneous development of autoimmune diabetes in NOD mice. To this end, 4-week-old NOD mice were treated with BMS309403 or vehicle by daily oral gavage for 8 weeks, followed by monitoring the incidence of diabetes until 30 weeks of age (Figure 3A). Treatment with BMS309403 did not affect food intake or physical activity (Supplemental Figure 2). The onset of diabetes occurred as early as 13 weeks old in vehicle-treated NOD mice, while it was postponed to approximately 20 weeks old in BMS309403-treated NOD (Figure 3A). At 30 weeks of age, approximately 88.2% of vehicle-treated NOD mice developed diabetes, whereas diabetes incidence of BMS309403-treated mice was significantly inhibited to 55.1% (Figure 3A). H&E staining analysis of the pancreatic sections showed that BMS309403 treatment preserved the islet mass and maintained the normal round shape compared with vehicle-treated mice, which was characterized by obvious infiltration of immune cells and islet destruction (Figure 3B). Insulitis scoring analysis showed that the number of islets with no insulitis (score = 0) was much higher in BMS-treated NOD mice (42% vs. 14%), and islets with more severe insulitis were inhibited significantly (score = 2, 18% vs. 31%; score = 3, 17% vs. 36%) compared with those of vehicle-treated NOD mice (Figure 3C). Moreover, to evaluate the effect of BMS309403 on β cell function in nondiabetic 16-week-old NOD mice (nonfasting blood glucose < 150 mg/dL), a glucose tolerance test (GTT) was performed, and the results showed that pharmacological inhibition of FABP4 significantly improved glucose tolerance capacity in NOD mice (Figure 3, D and E). Consistently, fasting insulin level in BMS309403-treated mice was also higher compared with that in vehicle-treated mice (Figure 3F), suggesting that pharmacological inhibition of FABP4 from an early age is sufficient to ameliorate β cell destruction and diabetes in NOD mice.

### Genetic ablation of FABP4 alleviates autoimmune diabetes in NOD mice.

**Figure 4:**
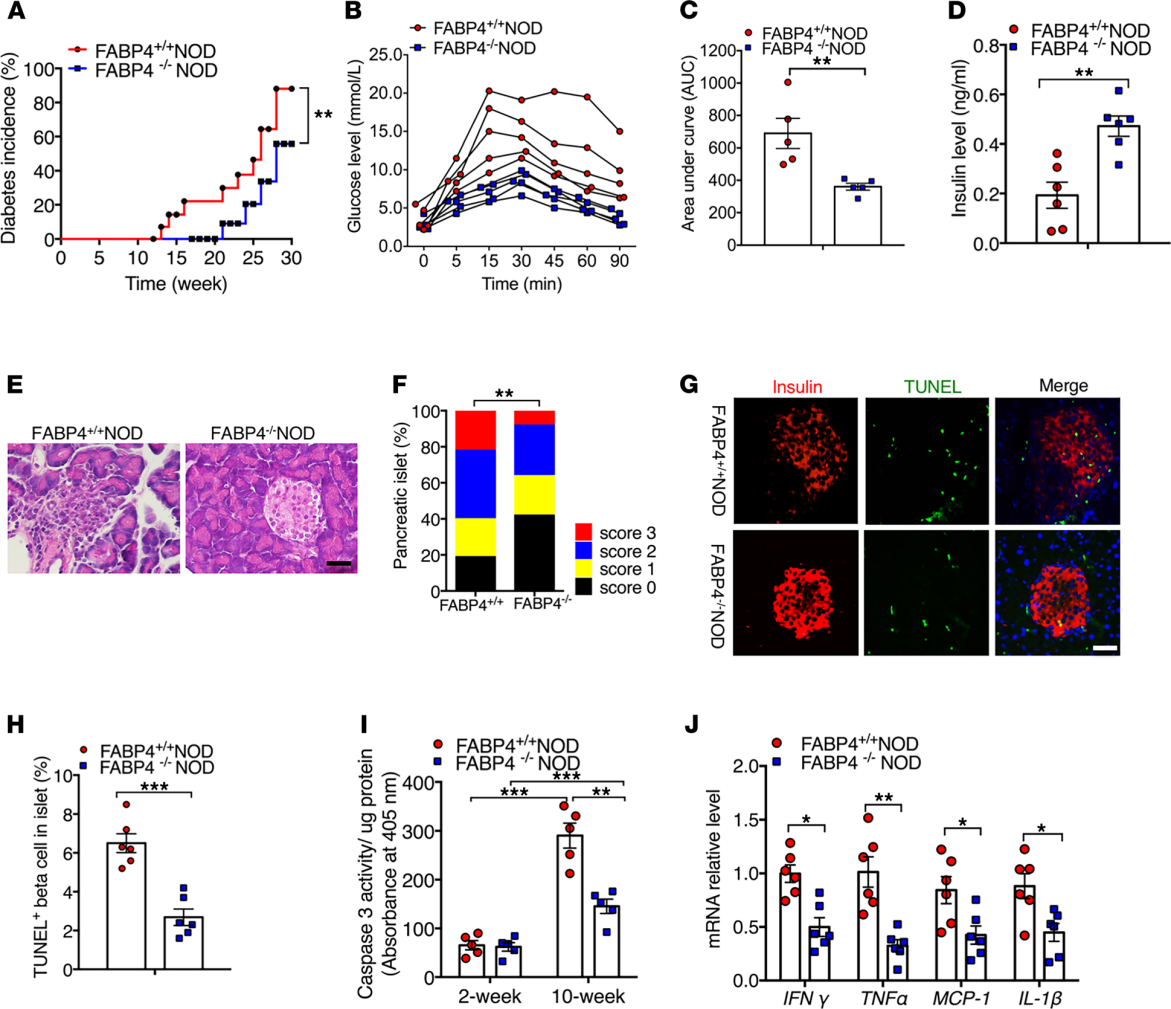
Genetic disruption of FABP4 alleviates autoimmune destruction of β cells and diabetes in NOD mice. (**A**) Incidence of diabetes in FABP4^+/+^NOD and FABP4^–/–^NOD mice at different ages. Diabetes incidence was diagrammed with the Kaplan-Meier method, and incidences between different groups were compared with the log-rank test (*n* = 18). (**B**) Glucose excursion curve for each individual mice after receiving glucose challenge, (**C**) the quantification of area under curve in glucose tolerance test, and (**D**) circulating insulin levels at 16 weeks of age (*n* = 6). (**E**) Representative images of H&E analysis for pancreatic sections and (**F**) insulitis scores in 10-week-old female FABP4^+/+^NOD and FABP4^–/–^NOD mice (*n* = 6). (**G**) Representative images of IHC staining of TUNEL (red) and insulin (green) in pancreases of 10-week-old female FABP4^+/+^NOD and FABP4^–/–^NOD mice. Scale bar: 20 mm, with original magnification of 400× (*n* = 6). (**H**) The quantification of absolute TUNEL-positive β cells in islets (*n* = 6). (**I**) Caspase-3 activity in islets lysate of 2-week- and 10-week-old FABP4^+/+^NOD and FABP4^–/–^NOD mice measured by Caspase-3 Fluorometric Assay Kit (*n* = 6). (**J**) The relative mRNA abundance of inflammatory cytokines (*IFNγ*, *TNFα*, *IL1β*, and *MCP1*) in the islets of FABP4^+/+^NOD and FABP4^–/–^NOD mice (*n* = 6). Data are expressed as mean ± standard deviation. Statistical significance was determined by 1-way analysis of variance or Student’s *t* test. *P < 0.05, **P < 0.01, ***P < 0.001.

To further investigate whether elevated FABP4 is involved in autoimmune diabetes, we crossed FABP4 knockout (FABP4^–/–^) with NOD/ShiLtJ mice for at least 10 generations to obtain FABP4^+/+^NOD and FABP4^–/–^NOD mice. The genotypes of both mouse strains were confirmed by real-time PCR and immunoblotting analyses (Supplemental Figure 3, A and B), confirming the successful ablation of FABP4 in FABP4^–/–^NOD mice. Diabetes incidence was regularly monitored in both FABP4^+/+^NOD and FABP4^–/–^NOD mice from 8 weeks old to 30 weeks old. The onset of diabetes occurred as early as 13 weeks old in FABP4^+/+^NOD mice, while it was postponed to 21 weeks in FABP4^–/–^NOD mice (Figure 4A). At 30 weeks of age, diabetes incidence reached 87.5% in FABP4^+/+^NOD mice but was significantly inhibited to 55.8% in FABP4^–/–^NOD mice (Figure 4A). FABP4^–/–^NOD mice also displayed better glucose tolerance in response to oral glucose challenge (Figure 4, B and C) and higher insulin secretion than FABP4^+/+^NOD mice (Figure 4D). H&E staining analysis of the pancreatic sections showed more severe islet destruction and much higher score of insulitis in FABP4^+/+^NOD mice than in FABP4^–/–^NOD mice (Figure 4, E and F). In situ cell death detection with TUNEL staining revealed that FABP4 deficiency significantly reduced β cell apoptosis in islets of NOD mice (Figure 4, G and H), and this was confirmed by measurement of cleaved caspase-3 activity in islets of FABP4^+/+^NOD and FABP4^–/–^NOD mice (Figure 4I). The gene expression of several inflammatory cytokines, including *IFNγ*, *TNFα*, monocyte chemoattractant protein–1 (*MCP1*), and *IL1β*, were significantly decreased in the pancreatic islets of FABP4^–/–^NOD compared with those in FABP4^+/+^NOD mice (Figure 4J), Taken together, these findings suggest the possible involvement of FABP4 in triggering the spontaneous development of insulitis, β cell self-destruction, and autoimmune diabetes in NOD mice.

### FABP4 deficiency reduces diabetogenic T cells and inflammatory macrophages.

**Figure 5:**
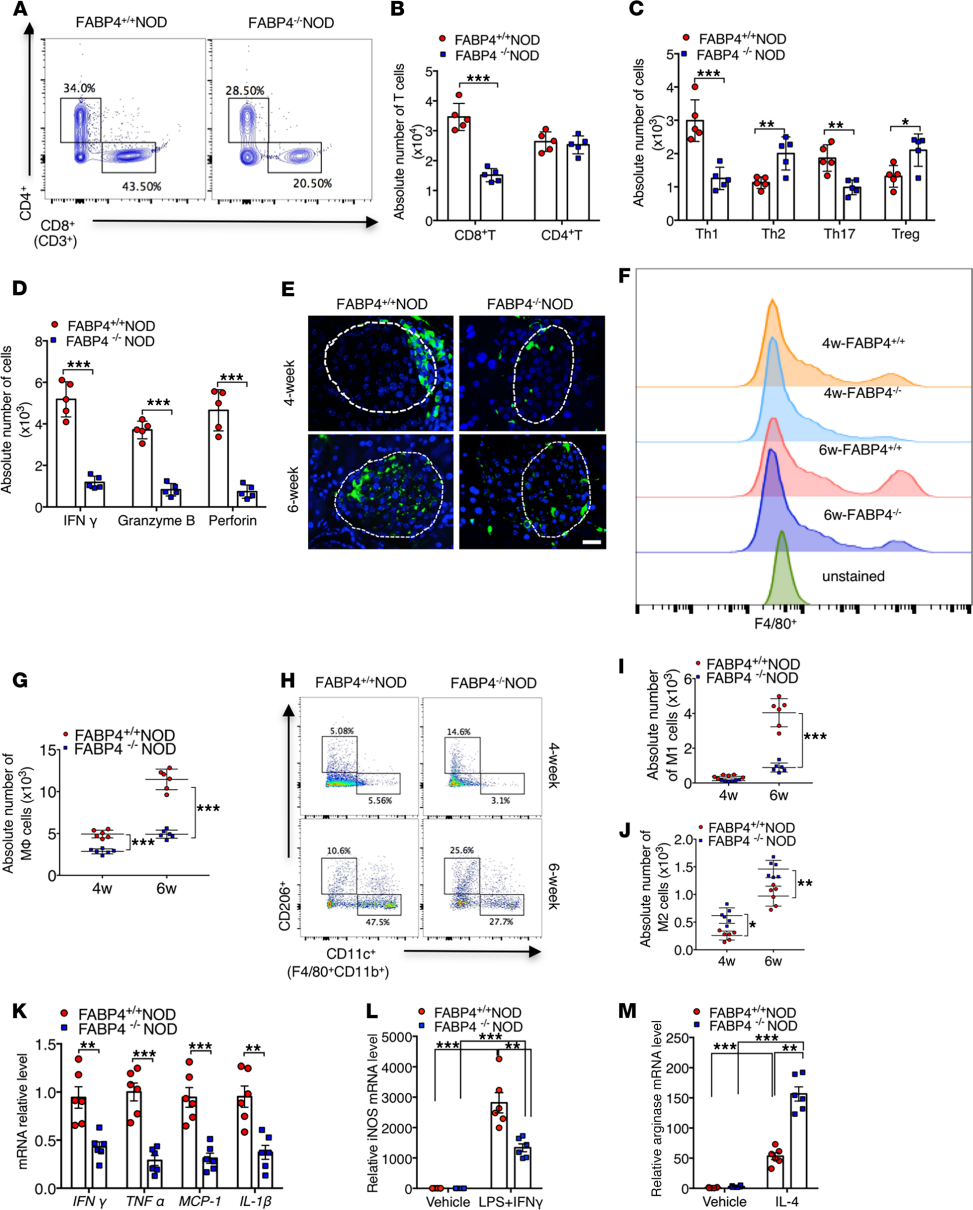
FABP4 deficiency reduces diabetogenic T cells and inflammatory macrophages in pancreatic islets of NOD mice. (**A**) Representative FACS plots showing the frequencies of cytotoxic T cells and helper T cells in total T cells from islets of 10-week-old FABP4^+/+^NOD and FABP4^–/–^NOD mice. (**B**) Quantification of the absolute numbers of cytotoxic T cells and helper T cells in total T cells from islets (*n* = 5). (**C**) Quantification of the absolute numbers of Th1, Th2, Th17, and Treg cells in helper T cells from islets (*n* = 5). (**D**) Quantification of the absolute number of IFN-γ, perforin, and granzyme B in cytotoxic T cells from islets (*n* = 5). (**E**) Representative images of IHC staining of macrophages (F4/80^+^, green; DAPI, blue) in islets of 4-week-old and 6-week-old FABP4^+/+^NOD and FABP4^–/–^NOD mice. Scale bar: 20 μm, with original magnification of 400× (*n* = 6). (**F**) Infiltration of macrophage (MΦ) among CD45^+^cells in islets of 4-week-old and 6-week-old FABP4^+/+^NOD and FABP4^–/–^NOD mice. (**G**) Quantification of absolute number of macrophages (MΦ) among CD45^+^ cells from islets (*n* = 6). (**H**) Representative FACS plots showing the absolute number of M1 and M2 macrophages from islets in 4-week-old and 6-week-old FABP4^+/+^NOD and FABP4^–/–^NOD mice. (**I** and **J**) The quantification of the absolute number of (**I**) M1 or (**J**) M2 macrophages in total macrophages from islets (*n* = 6). (**K**) The relative mRNA abundance of inflammatory cytokines in macrophages sorted from the pancreases of mice (*n* = 6). (**L** and **M**) Bone marrow–derived macrophages from FABP4^+/+^NOD or FABP4^–/–^NOD mice were treated with LPS (10 ng/mL) + IFN-γ (100 ng/mL) to induce M1 polarization or IL-4 (10 ng/mL) to induce M2 polarization. The mRNA abundance of (**L**) *iNOS* or (**M**) *arginase* was determined by real-time PCR analysis (*n* = 6). Data are expressed as mean ± standard deviation. Statistical significance was determined by 1-way analysis of variance or Student’s *t* test. *P < 0.05, **P < 0.01, ***P < 0.001.

Autoreactive T cells, including CD8^+^ cytotoxic T lymphocytes (CD8^+^CTLs) and CD4^+^ helper T lymphocytes (CD4^+^Th), are directly responsible for autoimmune destruction of β cells in type 1 diabetes (25). Therefore, we next compared the pancreatic abundance of these T cells between FABP4^+/+^NOD and FABP4^–/–^NOD mice with flow cytometry analysis. There was no obvious difference in total CD4^+^Th lymphocytes between 10-week-old FABP4^+/+^NOD and FABP4^–/–^NOD mice (Figure 5, A and B, and Supplemental Figure 4A). However, FABP4^–/–^NOD mice exhibited significantly lower frequency of Th1 (IFN-γ^+^CD4^+^) and Th17 (IL-17^+^CD4^+^) but higher abundance of Th2 (IL-4^+^CD4^+^) and Treg (Foxp3^+^CD4^+^) cells when compared with FABP4^+/+^NOD mice, suggesting that FABP4 deficiency shifts CD4^+^Th cells from Th1 to Th2 subtypes (Figure 5C and Supplemental Figure 4B). Furthermore, the abundance of CD8^+^CTLs in the islets of FABP4^–/–^NOD mice was significantly lower than that in FABP4^+/+^NOD mice (Figure 5, A and B), accompanied with reduced expression levels of IFN-γ, perforin, and granzyme B (Figure 5D and Supplemental Figure 4C), which reflect the cytotoxicity and penetrability of CTLs (26). Taken together, these findings suggest that FABP4 deficiency limits the formation of diabetogenic effector T cells.

As FABP4 is mainly expressed in pancreatic macrophages of NOD mice at an early age (Figure 2), and infiltrated macrophages play a key role in initiation of autoimmune attack of β cells (27), we next investigated the impact of FABP4 deficiency on number and phenotype of pancreatic macrophages in NOD mice. Immunohistochemistry staining analysis demonstrated that macrophages were mainly present at peri-islet sites at 4 weeks old but had penetrated into intra-islet sites in 6-week-old FABP4^+/+^NOD mice (Figure 5E). The number of infiltrated macrophages in the pancreatic islets of both 4-week-old and 6-week-old FABP4^–/–^NOD mice were significantly decreased when compared with age-matched FABP4^+/+^NOD mice. Consistently, flow cytometry analysis also showed much lower abundance of F4/80^+^ macrophages in the pancreas of FABP4^–/–^NOD mice than in FABP4^+/+^NOD mice at both 4 weeks and 6 weeks of age (Figure 5, F and G, and Supplemental Figure 5A).

Since proinflammatory M1 (F4/80^+^CD11b^+^CD11c^+^) and antiinflammatory M2 (F4/80^+^CD11b^+^CD206^+^) macrophages play opposite roles in the pathogenesis of autoimmune diabetes (28), we further investigated the effect of FABP4 on macrophage polarization in islets of NOD mice. Both 4-week-old and 6-week-old FABP4^–/–^NOD mice displayed much lower frequency of M1 macrophages, but higher abundance of M2 macrophages, than age-matched FABP4^+/+^NOD mice (Figure 5, H–J). The mRNA abundance of several proinflammatory cytokines (*TNFα*, *MCP1*, and *IL1β*) in macrophages sorted from the pancreas of FABP4^–/–^NOD mice was significantly reduced compared with that from FABP4^+/+^NOD mice (Figure 5K). Consistently, bone marrow–derived FABP4^–/–^ macrophages exhibited a marked reduction of IFN-γ/LPS–induced expression of *iNOS* (a marker for M1 macrophages) but an obvious elevation of IL-4–induced expression of the M2 macrophage *arginase* (Figure 5, L and M). On the other hand, there was no obvious difference in the abundance of pancreatic DCs, neutrophils, and NK cells between FABP4^–/–^NOD and FABP4^+/+^NOD mice (Supplemental Figure 5). Collectively, these data suggest that elevated FABP4 contributes to both infiltration and proinflammatory polarization of macrophages in juvenile NOD mice.

### Macrophagic FABP4 contributes to autoimmune destruction of β cells and diabetes in NOD mice.

**Figure 6:**
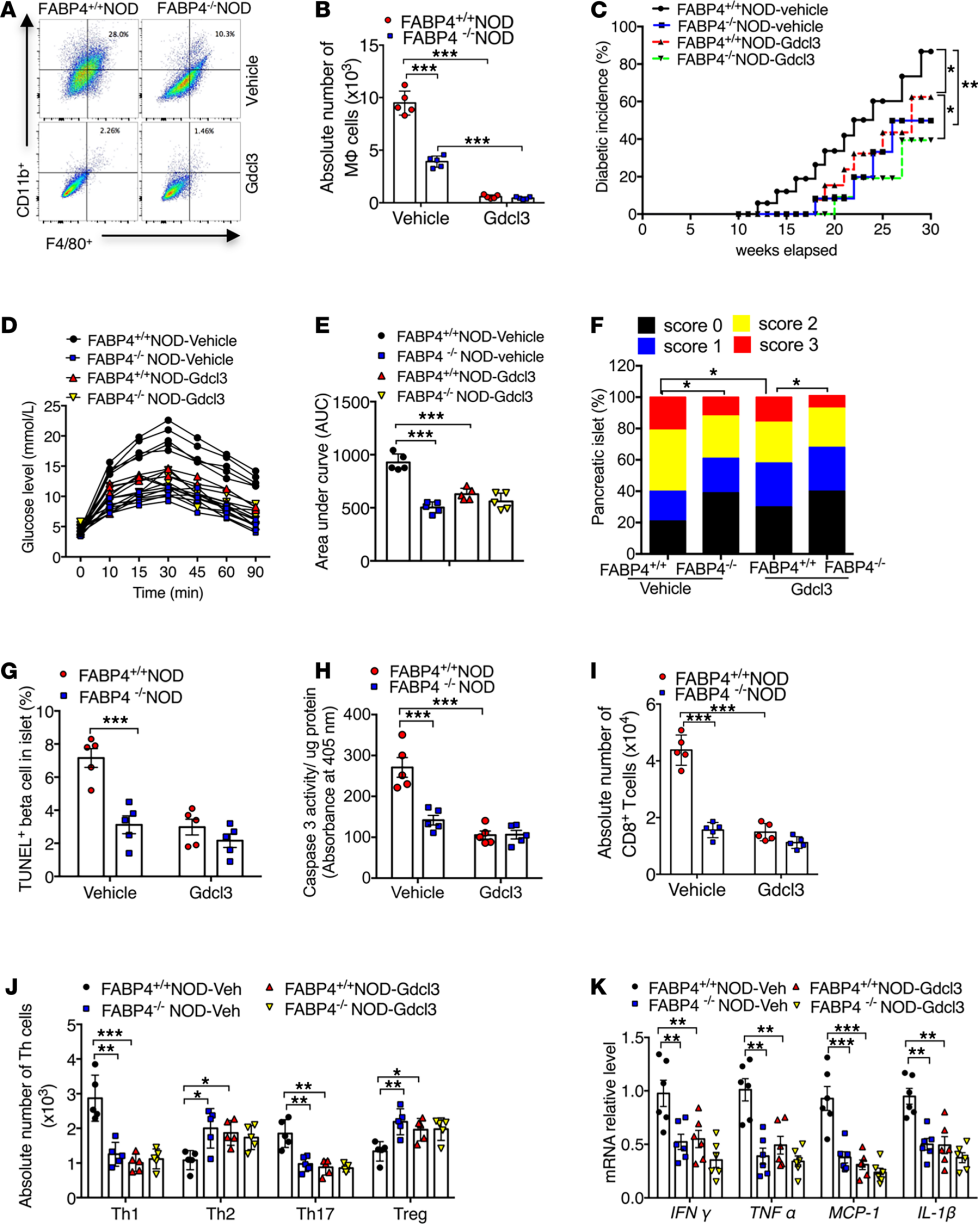
Macrophage-expressed FABP4 contributes to the development of insulitis and diabetes in NOD mice. Two-week-old female FABP4^+/+^NOD mice and FABP4^–/–^NOD mice were intravenously injected with GdCl_3_ (1 mg/kg) or saline every 3 days (i.v.) for 6 weeks. Diabetes incidence was monitored in the following 30 weeks. (**A**) Infiltration and (**B**) quantification of F4/80^+^ macrophages among CD45^+^ cells in islets of NOD mice treated with GdCl_3_ or vehicle for 6 weeks (*n* = 5). (**C**) Diabetes incidence in FABP4^+/+^NOD mice and FABP4^–/–^NOD mice treated with GdCl_3_ or vehicle. Diabetes incidence was diagrammed with the Kaplan-Meier method, and incidences between different groups were compared with the log-rank test (*n* = 18 in each group). (**D**) Glucose tolerance test of 14-week-old FABP4^+/+^NOD and FABP4^–/–^NOD mice treated with GdCl3 and vehicle control. (**E**) The quantification of area under curve of different groups (*n* = 5). (**F**) Insulitis scores determined by histological evaluation of pancreatic sections as in Figure 4B (*n* = 6). (**G**) Quantification of TUNEL-positive β cells in islets of 10-week-old FABP4^+/+^NOD mice and FABP4^–/–^NOD mice (*n* = 5). (**H**) Cleaved caspase-3 activity in pancreatic lysate of FABP4^+/+^NOD mice and FABP4^–/–^NOD mice treated with GdCl_3_ or vehicle for 6 weeks measured by Caspase-3 Fluorometric Assay Kit (BioVision, Inc.) (*n* = 5). (**I**) Quantification of the absolute number of CD8^+^ T cells in total T cells (CD3^+^) from islets of above mice (*n* = 5). (**J**) Quantification of the absolute numbers of Th1 (IFN-γ^+^CD4^+^), Th2 (IL-4^+^CD4^+^), Th17 (IL-17^+^CD4^+^), and Treg (Foxp3^+^CD4^+^) cells in total helper T cells (CD4^+^CD3^+^) from islets of above mice (*n* = 5). (**K**) The relative mRNA abundance of inflammatory cytokines (*IFNγ*, *TNFα*, *IL1β*, and *MCP1*) in macrophages sorted from the pancreases of above mice treated with GdCl_3_ or vehicle (*n* = 6). Data are expressed as mean ± standard deviation. Statistical significance was determined by 1-way analysis of variance or Student’s *t* test. *P < 0.05, **P < 0.01, ***P < 0.001.

To investigate the pathogenic roles of macrophage-expressed FABP4 in autoimmune diabetes, we first depleted macrophages in FABP4^+/+^NOD and FABP4^–/–^NOD mice by injection with GdCl_3_ (1 mg/kg) or vehicle (PBS) every 3 days from 2 weeks to 8 weeks of age (Figure 6, A and B). The efficiency of macrophage depletion was confirmed by flow cytometry analysis showing that only 2%~3% pancreatic macrophages remained after 6 weeks of GdCl_3_ treatment compared with vehicle-treated mice (Figure 6, A and B). Notably, macrophage depletion in FABP4^+/+^NOD mice markedly delayed the onset time from 12 weeks to 18 weeks and also significantly reduced the incidence of diabetes from 86.7% to 62.3% at 30 weeks of age (Figure 6C). Glucose tolerance capacity was also significantly improved in macrophage-depleted FABP4^+/+^NOD mice compared with vehicle-treated controls (Figure 6, D and E). On the other hand, depletion of macrophages only led to a modest reduction in the incidence rate of diabetes (39.4% in GdCl_3_-treated vs. 49.8% in vehicle-treated group) and a slight improvement in glucose tolerance in FABP4^–/–^NOD mice (Figure 6, C–E).

Macrophage depletion in FABP4^+/+^NOD mice caused a marked reduction in insulitis score (Figure 6F) and in β cell apoptosis, as determined by immunocytochemistry and TUNEL (Figure 6G) and biochemical analysis of cleaved caspase-3 activity in pancreatic islets (Figure 6H). These changes in macrophage-depleted FABP4^+/+^NOD mice were accompanied by significantly reduced number of CD8^+^CTLs (Figure 6I), increased frequencies of Th2 and Tregs and decreased abundance of Th1 and Th17 (Figure 6J), and lower expression of proinflammatory cytokines compared with the pancreatic tissue of vehicle-treated FABP4^+/+^NOD mice (Figure 6K). On the other hand, the effects of macrophage depletion on alleviation of insulitis, β cell apoptosis, autoreactive T cells, and inflammatory cytokines in FABP4^–/–^NOD mice were much less obvious than those observed in FABP4^+/+^NOD mice. Taken together, these findings suggest that reduced incidence of autoimmune diabetes in FABP4^–/–^NOD mice can be partly explained by FABP4 deficiency in macrophages.

### Transplantation of FABP4-deficient bone marrow cells reduces insulitis and autoimmune diabetes in NOD mice.

**Figure 7:**
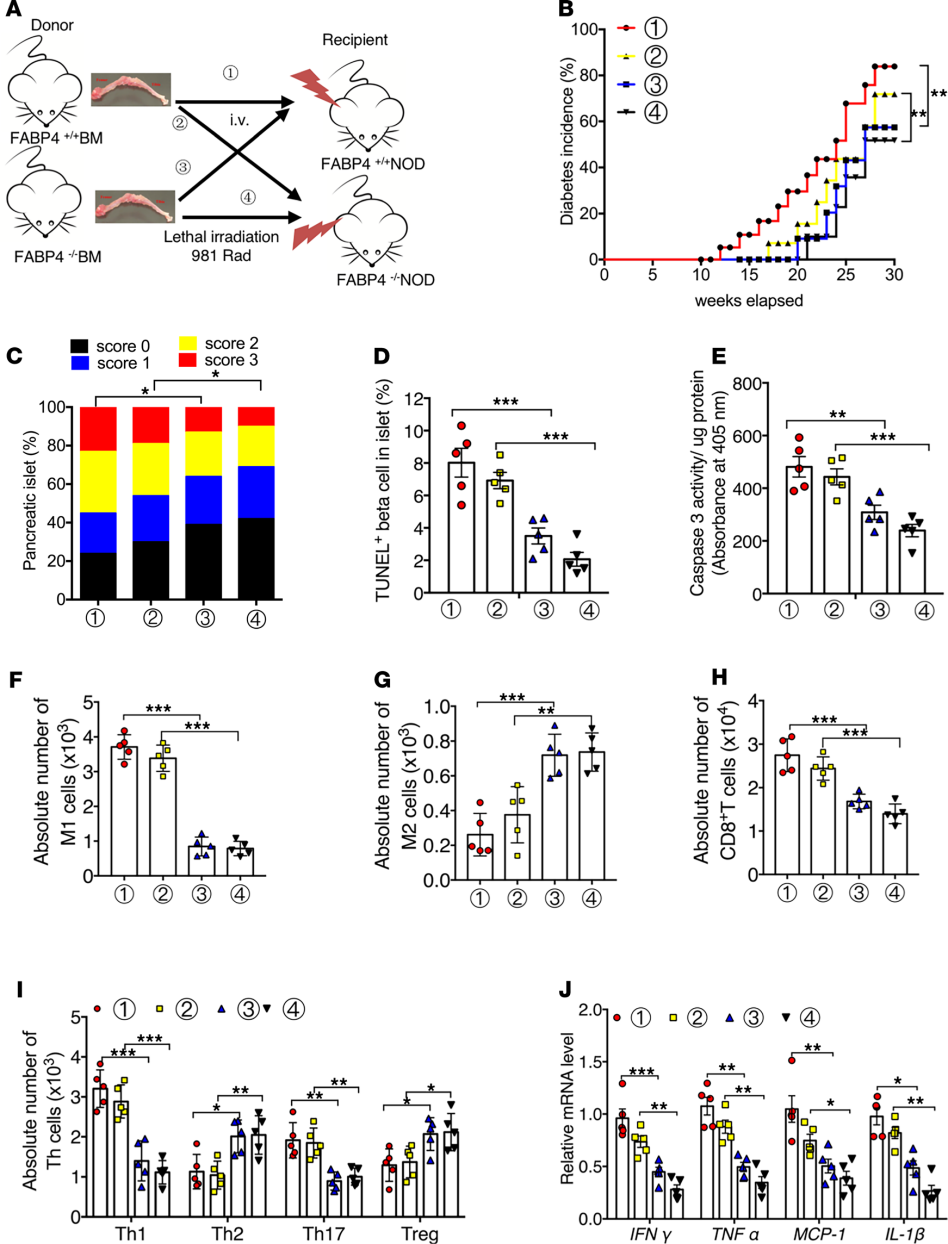
Adoptive transfer of FABP4-deficient BM cells ameliorates insulitis and autoimmune diabetes in NOD mice. (**A**) Schematic diagram showing the protocol of bone marrow transplantation (BMT). 1. donor: FABP4^+/+^BM→recipient: FABP4^+/+^NOD mice. 2. donor: FABP4^+/+^BM→recipient: FABP4^–/–^NOD mice. 3. donor: FABP4^–/–^BM→recipient: FABP4^+/+^NOD mice. 4. donor: FABP4^–/–^BM→recipient: FABP4^–/–^NOD mice. (**B**) Diabetes incidence in these mice at different ages after BMT. Diabetes incidence was diagrammed with the Kaplan-Meier method, and incidences between different groups were compared with the log-rank test (*n* = 18 in each group). (**C**) Calculated insulitis scores in each group based on 100 islets from each group (*n* = 5). (**D**) Quantification of TUNEL-positive β cells in islets of recipient NOD mice (*n* = 5). (**E**) Caspase-3 activity in pancreatic lysates measured by Caspase-3 Fluorometric Assay Kit (*n* = 5). (**F** and **G**) Quantification of the absolute number of (**F**) M1 macrophages (CD11c^+^CD11b^+^F4/80^+^) and (**G**) M2 macrophages (CD206^+^CD11b^+^F4/80^+^) in total macrophages (MΦ, CD11b^+^F4/80^+^) from pancreatic islets of recipient mice (*n* = 5). (**H**) Quantification of the absolute number of CD8^+^ T cells in total T cells (CD3^+^) from islets of recipient mice. (**I**) Quantification the absolute number of Th1 (IFN-γ^+^CD4^+^), Th2 (IL-4^+^CD4^+^), Th17 (IL-17^+^CD4^+^), and Treg (Foxp3^+^CD4^+^) cells in total helper T cell (CD4^+^CD3^+^) from islets of recipient mice (*n* = 5). (**J**) The relative mRNA abundance of inflammatory cytokines (*IFNγ*, *TNFα*, *IL1β*, and *MCP1*) in the islets of recipient mice (*n* = 5). Data are expressed as mean ± standard deviation. Statistical significance was determined by 1-way analysis of variance or Student’s *t* test. *P < 0.05, **P < 0.01, ***P < 0.001.

To further dissect the contribution of macrophage-expressed FABP4 in autoimmune destruction of β cells and diabetes, we next performed adoptive transfer experiments by transplantation of FABP4^–/–^ and FABP4^+/+^ bone marrow (BM)into FABP4^+/+^NOD and FABP4^–/–^NOD mice, respectively (Figure 7A). FABP4^+/+^NOD mice transplanted with FABP4^–/–^ BM exhibited much lower incidence of diabetes than FABP4^+/+^NOD mice transplanted with FABP4^+/+^ BM (57.38% vs. 83.91%) (Figure 7B), accompanied with significant reductions in insulitis (Figure 7C), β cell apoptosis (Figure 7, D and E), proinflammatory macrophages (Figure 7, F and G), autoreactive CD8^+^ T cells, Th1, and Th17, but increased frequencies of Th2 and Tregs (Figure 7, H and I). Furthermore, reconstitution of FABP4^+/+^NOD mice with FABP4^–/–^ BM markedly decreased the expression of a panel of inflammatory cytokines in pancreatic islets (Figure 7J). In contrast, transplantation of FABP4^+/+^ BM into FABP4^–/–^NOD mice significantly increased the incidence of diabetes (71.86% vs. 51.79%) and aggravated insulitis, β cell apoptosis, inflammation, and activation of autoreactive T cells compared with FABP4^–/–^NOD mice transplanted with FABP4^–/–^ BM (Figure 7).

To explore whether FABP4 influences the development of autoimmune diabetes via its actions in diabetogenic T cells, we isolated CFSE-labeled CD4^+^ T or CD8^+^ T cells from FABP4^–/–^NOD and FABP4^+/+^NOD mice and adoptively transferred these diabetogenic T cells into FABP4^–/–^NOD mice, followed by monitoring diabetes incidence in recipient mice (Supplemental Figure 6, A–C). Flow cytometry analysis in pancreatic lymph nodes confirmed the successful transfer of CFSE-labeled CD4^+^ T or CD8^+^ T cells into FABP4^–/–^NOD mice (Supplemental Figure 6D). In line with previous reports (29–32), adoptive transfer of both diabetogenic CD4^+^ T and CD8^+^ T cells from both types of donor mice (FABP4^–/–^NOD and FABP4^+/+^NOD mice) into FABP4^–/–^NOD mice accelerated the development of autoimmune diabetes, as evidenced by much earlier onset and higher incidence of diabetes compared with FABP4^–/–^NOD mice treated with vehicle control (Supplemental Figure 6E). However, the diabetogenic effects of these T cells derived from FABP4^–/–^NOD and FABP4^+/+^NOD mice were comparable, further confirming the notion that FABP4 has no direct effect on diabetogenic T cells. Taken together, these data further support the notion that the diabetogenic effect of FABP4 in NOD mice is partly attributed to its expression and actions in macrophages.

## Discussion

Despite the fact that the diabetogenic T cells are the direct contributor to autoimmune destruction of β cells in type 1 diabetes, there is increasing evidence that innate immune cells are the key players in the early pathogenesis of this disease (33). Infiltration and activation of neutrophils, macrophages, and NK cells in pancreatic islets of NOD mice can be detected before T cell infiltration. However, the pathological factors triggering the recruitment and/or activation of these innate immune cells in pancreatic islets remain largely unknown. In the present study, we identified FABP4 as an early mediator in the pathogenesis of type 1 diabetes through its actions in islet macrophages. In humans, circulating FABP4 level is elevated in patients with type 1 diabetes as well as their FDRs and is closely associated with the titers of islet autoantibodies. In animals, increased FABP4 in pancreatic islets and circulation of NOD mice occurs as early as 4 weeks of age, well before the development of overt diabetes. Pharmacological inhibition or genetic ablation of FABP4 at an early age is sufficient to reduce autoimmune destruction of β cells and subsequent development of diabetes.

The pathogenic role of macrophages in the development of insulitis and type 1 diabetes has been well documented in several previous studies (27, 34, 35). The spontaneous development of insulitis and autoimmune diabetes is markedly attenuated by inhibition of macrophage influx into the pancreatic islets with a monoclonal antibody that blocks an adhesion-promoting receptor (36) or by macrophage depletion with clodronate-loaded liposomes (9). Macrophages not only induce β cell damage or death by releasing TNF-α, IL-1β, and ROS but also promote efficient differentiation of diabetogenic CD8^+^CTLs leading to type 1 diabetes onset (27). Furthermore, the chemokines C-X-C motif chemokine ligand 1 (CXCL1) and CXCL2 produced from macrophages play an obligatory role in recruiting C-X-C motif chemokine receptor 2–expressing neutrophils from the blood to the pancreatic islets (34). In this connection, our present study provides several lines of evidence supporting the notion that the effects of FABP4 in promoting insulitis and autoimmune diabetes are attributed to its expression and actions in macrophages. FABP4 was predominantly expressed in macrophages but not in other types of immune cells in the pancreatic islets of NOD mice (Figure 2). FABP4^+/+^NOD mice were much more susceptible to developing insulitis and autoimmune diabetes than FABP4^–/–^NOD mice, whereas such a difference between these 2 types of mice was largely diminished after depletion of macrophages (Figure 6). Furthermore, adoptive transfer of FABP4^–/–^ BM cells into FABP4^+/+^NOD mice reduced insulitis and autoimmune diabetes, while transplantation of FABP4^+/+^ BM cells into FABP4^–/–^NOD mice caused the opposite changes (Figure 7). Notably, FABP4 deficiency not only decreased the total number of infiltrated macrophages but also promoted macrophage polarization from classically activated proinflammatory M1 phenotype to alternatively activated antiinflammatory M2 phenotype. In line with our findings, a single adoptive transfer of M2 macrophages has been shown to protect 80% of NOD mice from developing type 1 diabetes for at least 3 months (28).

The proinflammatory activity of FABP4 in macrophages has been reported previously in the context of obesity, atherosclerosis, and nonalcoholic fatty liver disease (15, 18, 37). Genetic ablation or pharmacological inhibition of FABP4 in macrophages attenuates NF-κB signaling (38) and blocks LPS-induced activation of c-Jun N-terminal kinase (JNK) and secretion of inflammatory cytokines (39). Notably, FABP4 serves as a downstream effector of Toll-like receptors (TLRs) and plays an obligatory role in mediating LPS-induced inflammatory responses in macrophages. FABP4 expression in macrophages is elevated by 1500-fold, 9-fold, and 56-fold respectively in response to stimulation with agonists of TLR2, 3, and 4, respectively (40). Loss of FABP4 leads to a marked attenuation in TLR4 agonist–induced activation of NF-κB and JNK and production of proinflammatory cytokines (39, 41). As the key components in the innate immune system, TLRs play a crucial role in the pathogenesis of type 1 diabetes by inducing the maturation of APCs and by stimulating the production of inflammatory chemokines and cytokines (42). Targeting innate immunity in NOD mice with anti-TLR4/MD2 antibodies effectively reverses type 1 diabetes by downmodulating adaptive immunity (43). Taken together, these findings suggest that augmented TLR/FABP4 signaling axis might be an important link between innate and adaptive immune crosstalk during the onset and progression of insulitis and type 1 diabetes.

Although our results showed that FABP4 deficiency led to reduced number of CD8^+^CTLs and the shift of CD4^+^Th lymphocytes from Th1 to Th2 subtypes (Figure 5), FABP4 was not expressed in these T cells. Therefore, it is likely that the changes of these diabetogenic T lymphocytes in FABP4^–/–^NOD mice are not due to the direct effects of FABP4 in these cells but are secondary to alterations of FABP4-deficient macrophages. This notion is further supported by our adoptive cell transfer experiments showing that transplantation of FABP4-deficient BM cells into FABP4^+/+^NOD mice was sufficient to decrease the frequency of CD8^+^ T lymphocytes and to promote the shift of CD4^+^Th lymphocytes from Th1 to Th2 subtypes, whereas reconstitution of FABP4^–/–^NOD mice with FABP4^+/+^ BM cells led to the opposite changes in these T cells. Consistent with our findings, T cells in a macrophage-depleted environment lost their ability to differentiate into β-cytotoxic T cells, but these T cells regained their β cell–cytotoxic potential when returned to a macrophage-containing environment (27). Macrophage depletion in NOD mice led to a decrease in Th1 but increase in Th2 immune responses. Macrophages have been shown to promote efficient differentiation of diabetogenic T cells by secreting IL-12 (33), which was obviously reduced in FABP4-deficient macrophages (Figure 5). Although our current study focused on the role of FABP4 in infiltration and activation of macrophages in pancreatic islets, it remains possible that the modulatory effect of FABP4 on macrophages in other organs, such as liver and gut, may also contribute to the pathogenesis of type 1 diabetes by altering the tolerogenic state. Altered mucosal immune system in the gut is an important contributor to the failure to form tolerance, resulting in the autoimmunity that underlies type 1 diabetes (44). Moreover, dysregulated innate immunity is a common early feature in development of type 1 diabetes that affects metabolic homeostasis and tolerogenic phenotype in the prediabetic liver of NOD mice (45).

Due to the critical roles of FABP4 in immunometabolism, a large number of highly specific chemical inhibitors of FABP4 have been developed as potential drug candidates for treatment of metabolic and inflammatory diseases (13, 41). Several FABP4 inhibitors have been shown to be effective in alleviating chronic inflammatory disorders, including insulin resistance, atherosclerosis, nonalcoholic fatty liver disease, and osteoarthritis (17, 18, 46). Our results demonstrated that treatment of NOD mice with the FABP4 inhibitors at an early age was sufficient to reduce the later development of autoimmune diabetes, suggesting that the therapeutic applications of the FABP4 inhibitors can be expanded into management of autoimmune diseases.

## Methods

### Human study.

All participants were recruited from The Second Xiangya Hospital, Central South University. A total of 92 type 1 diabetes patients who had been diagnosed with diabetes for less than 1 year and 93 FDRs (parents, siblings, and offspring) of type 1 diabetes patients were recruited for this study. Patients with type 1 diabetes were diagnosed according to the criteria of the American Diabetes Association (47). All patients were treated with insulin. One hundred two healthy control subjects were recruited using the following inclusion criteria: NGT with fasting plasma glucose of less than 5.6 mmol/L and 2-hour plasma glucose of less than 7.8 mmol/L by oral glucose tolerance test (OGTT) and no family history of diabetes or other autoimmune diseases. All FDRs of type 1 diabetes patients had NGT confirmed by OGTT and were screened for autoantibodies against GAD65, IA-2A, and ZnT8.

The exclusion criteria for all participants were as follows: (a) presence of other autoimmune diseases; (b) use of glucocorticoids and/or immunomodulators in the past 1 year; (c) presence of heart, liver, kidney, and other important organ failures and malignant diseases; (d) for pregnant and lactating women, gestational diabetes, or secondary diabetes; (e) acute stress or acute trauma, surgery, and other acute stress states in the past 2 weeks.

### Clinical and biochemical assessments.

For all participants, height, weight, blood pressure, waist circumference, and hip circumference were measured with a standardized procedure. BMI and weight/hip ratio were calculated based on those measurements.

Venous blood samples were drawn at fasting state and 2 hours after a meal in separation gel coagulation promoting tubes for serum samples and in anticoagulation tubes for plasma samples. After standing still at room temperature for 2 hours, serum samples were collected after centrifugation at 2000*g* for 10 minutes. Plasma glucose level was analyzed by Hitachi 7170 analyzer (Boehringer Mannheim). Serum cholesterol, triglycerides, high-density lipoprotein–cholesterol, and low-density lipoprotein–cholesterol levels were measured by enzymatic assays. Serum levels of C-peptide were measured using the Advia Centaur System (Siemens). Glycated hemoglobin A1c was detected by ion exchange high-performance liquid chromatography (HLC-723G8, Tosoh), and intra-assay and inter-assay differences were less than 1% and less than 3%, respectively. GADA, IA-2A, and ZnT8A autoantibodies were detected by the radioligand assay with in vitro–translated ^35^S-methionine–labeled GAD65, IA-2A, or ZnT8 as we described previously (48–50). In the 2016 Islet Autoantibody Standardization Program, the sensitivity and specificity in our laboratory were 82% and 97.8% for GADA, 76% and 100% for IA-2A, and 72% and 100% for ZnT8A. The inter-assay coefficients of variation (CV) of GADA, IA-2A, and ZnT8A were 7.1%–10.8%, 3.2%–9.7%, and 3.9%–9.8%, respectively, and the intra-assay CV of GADA, IA-2A, and ZnT8A were 4.9%–8.3%, 5.6%–11.7%, and 4.3%–13.8%, respectively. The fasting serum FABP4 concentration of the subjects was measured by enzyme-linked immunosorbent assays (Immunodiagnostics). The intra-assay and inter-assay CV for the FABP4 assay were less than 4.1% and less than 5.5%, respectively.

### Isolation of human monocytes.

PBMCs from healthy controls and patients with type 1 diabetes were isolated by Ficoll density gradient centrifugation and washed twice with serum-free RPMI 1640 medium (Gibco, Thermo Fisher Scientific). PBMCs were then plated at 3 mL/well in 6-well cell culture plates for 4 hours. The medium and unbound cells were removed and adherent cells further cultured with RPMI 1640 complete medium (10% FBS, 1% penicillin/streptomycin). Cells were harvested, and RNA was extracted with RNAiso Plus (Takara) and reverse-transcribed into complementary DNA using PrimeScript RT Reagent Kit (Takara). Real-time PCR was performed using SYBR Green master mix (Takara) on a 7900HT (Applied Biosystems, Thermo Fisher Scientific). Primer sequences are listed in Supplemental Table 2.

### Animals.

NOD/ShiLtJ (namely NOD) mice, a polygenic model for autoimmune diabetes characterized by hyperglycemia and insulitis associated with leukocytic infiltration of the pancreatic islets, were bought from The Jackson laboratory (RRID: IMSR_ARC: NOD). Female NOD mice, which are more susceptible to spontaneous development of autoimmune diabetes (51), were used throughout this study. FABP4^–/–^ mice in C57BL/6N background were generated using the same procedures as previously described (52). NOR/LtJ (also known as NOR) mice are insulitis resistant and diabetes free, which matched at the diabetogenic H2g7 complex to NOD/ShiLtJ, served as controls to the NOD/ShiLtJ strain (RRID: MGI: 3582463). FABP4^–/–^ mice in C57BL/6N background were backcrossed with NOD/ShiLtJ mice for at least 10 generations to obtain FABP4^+/+^NOD mice and FABP4^–/–^NOD mice. Genotypes of these mice were confirmed using primers in Supplemental Table 1. Age-matched female FABP4^+/+^NOD mice and FABP4^–/–^NOD mice were used in all the experiments of this study. Animals were allocated to their experimental groups according to their genotypes. The investigators were not blinded to the experimental groups. Mice were housed in a temperature-controlled facility (23°C, 12-hour light/12-hour dark cycle, 60%–70% humidity) with free access to food (STC, Purina) and water for the entire experimental period. Key resources related to this study are listed in Supplemental Table 3.

### Diabetes diagnosis.

Nonfasting blood glucose concentrations of mice were measured by tail vein prick using a blood glucose test meter (ACCU-CHEK) and strips (Roche Diagnostics) according to manufacturer’s instruction every other day in the morning starting at 8 weeks of age. Any reading of blood glucose higher than 13.9 mmol/L (250 mg/dL) was confirmed by another test 24 hours later. Overt diabetes was diagnosed by 2 consensus positive blood glucose tests higher than 13.9 mmol/L.

### Isolation of pancreatic islets.

Pancreas from age-matched FABP4^+/+^NOD mice and FABP4^–/–^NOD mice were perfused with 3 mL of a solution containing Collagenase P (1 mg/mL, Roche Diagnostics), then digested for 10 minutes at 37°C. Digestion was stopped by adding HBSS–5% FBS followed by extensive washes. The homogenates were filtered through 500 μm and 70 μm cell strainers (BD Biosciences) sequentially, and islets captured in the 70 μm cell strainers were cultured in RPMI 1640 medium with 10% FBS (Invitrogen, Thermo Fisher Scientific) and 1% penicillin and streptomycin (Thermo Fisher Scientific) and further purified and counted by hand picking under a microscope (Bx41 System, Olympus).

### Flow cytometry analysis.

Single-cell suspensions were prepared from various tissues of age-matched FABP4^+/+^NOD mice and FABP4^–/–^NOD mice. Pancreatic islets isolated as described above were incubated in Cell Dissociation Solution Nonenzymatic (MilliporeSigma) at 37°C for 10 minutes prior to flow cytometry analysis. Cells were then harvested by centrifugation at 800*g* for 15 minutes at 4°C, resuspended in 1 mL of Live/Dead Fixable Dead Cell Stains (Molecular Probes, Thermo Fisher Scientific), incubated on ice for 30 minutes, and then washed with 1× PBS containing 1% BSA, followed by staining with different antibodies, as detailed in Supplemental Methods. After staining, cells were fixed with 2% (*w/v*) paraformaldehyde and stored at 4°C before analysis with BD LSR Fortessa Cell Analyzer (BD Biosciences). Data were analyzed using FlowJo software version X.0.7 (Tree Star, Inc.).

### BM transplantation.

Female 4-week-old FABP4^+/+^NOD and FABP4^–/–^NOD mice were provided with drinking water containing antibiotics (0.1 mg/mL neomycin; 0.01 mg/mL polymyxin B, MilliporeSigma) for 2 weeks and were then subjected to lethal irradiation at the age of 6 weeks with a dose of 981 cGy prior to receiving bone transplantation. BM cells were isolated from 5 age-matched FABP4^+/+^NOD and FABP4^–/–^NOD donor mice by flushing the humeral and femoral bones with sterile RPMI 1640 medium (Invitrogen, Thermo Fisher Scientific). Each recipient mouse was transfused with 5 million cells in 200 μL RPMI 1640 medium via tail vein injection. After a recovery period of 4 weeks, these recipient mice were used for monitoring diabetes incidence and insulitis.

### Glucose tolerance test.

Mice housed in clean cages were fasted for 16 hours before intraperitoneal injection with d-glucose (1 g/kg). One drop of blood was collected from tail veins of mice at 0, 10, 15, 30, 45, 60, 75, and 90 minutes after glucose challenge for measurement of glucose levels with blood glucose test meter (ACCU-CHEK) and strips (Roche Diagnostics) according to manufacturer’s instruction.

### Immunoassays, immunoblot analysis, and real-time PCR.

Serum concentrations of FABP4 (Biovendor) and insulin (Immunodiagnostics) in mice were determined with enzyme-linked immunosorbent assay kits, in-house or commercial, according to the manufacturer’s instructions, respectively.

Proteins were separated by SDS-PAGE, transferred to polyvinylidene difluoride membranes, and probed with primary antibodies against mouse FABP4 (0.25 mg/mL, goat polyclonal; catalog AF1443, R&D Systems, Bio-Techne), HSP90 (0.25 mg/mL, rabbit monoclonal; catalog 4877, Cell Signaling Technology). The intensities of protein bands were quantified using the NIH ImageJ software.

Total RNA was extracted with RNAiso Plus (Takara) and reverse-transcribed into complementary DNA using PrimeScript RT Reagent Kit (Takara). Real-time PCR was performed using SYBR Green master mix (Takara) on a 7900HT (Applied Biosystems, Thermo Fisher Scientific), normalized against the *GAPDH* gene. Primer sequences are listed in Supplemental Table 2.

### Statistics.

For clinical studies, all data analysis was performed using SPSS 25.0. Kolmogorov-Smirnov test was applied to test the distribution pattern. Data were expressed as the mean ± standard deviation or the median and IQR. Logarithmic transformations were applied for non-normally distributed parameters before statistical analysis. Differences between the groups were assessed by χ^2^ test for categorical data or unpaired Student’s *t* test for continuous variables. One-way ANOVA was used for comparisons among groups. Correlations were evaluated with Spearman or partial correlation as appropriate. In all statistical comparisons, 2-sided *P* < 0.05 was considered statistically significant.

For animal studies, diabetes incidence in NOD mice was diagrammed with the Kaplan-Meier method, and incidences between different groups were compared with the log-rank test. For other experiments, data are shown as mean values ± standard deviation. Statistical significance was determined by 1-way analysis of variance or 2-tailed Student’s *t* test or Gehan-Breslow-Wilcoxon testing of the Kaplan-Meier survival curve or Pearson’s χ^2^ test using V.19.0 of SPSS. *P* < 0.05 was considered statistically significant.

### Study approval.

The clinical trial was approved by the Human Ethics Committee of The Second Xiangya Hospital of Central South University. Informed consent was obtained from all participants in writing. The study was carried out in accordance with the principles of the Declaration of Helsinki.

For the animal experiments, all experimental protocols were approved by the Committee on the Use of Live Animals in Teaching and Research at the University of Hong Kong following the regulations established by the NIH’s *Guide for the Care and Use of Laboratory Animals* (National Academies Press, 2011).

## Author contributions

AX designed the study and wrote the manuscript. YX, LS, XW, YL, LYC, BL, and XX conducted the experiments and analyzed the data; YX and LS prepared the figures and drafted the manuscript; ZGZ coordinated clinical studies and edited the manuscript; and RLCH edited the manuscript. YX and LS contributed equally to this study: YX conducted the clinical and animal experiments, whereas LS conducted the animal and in vitro experiments.

## Supplementary Material

Supplemental data

## Figures and Tables

**Table 1:**
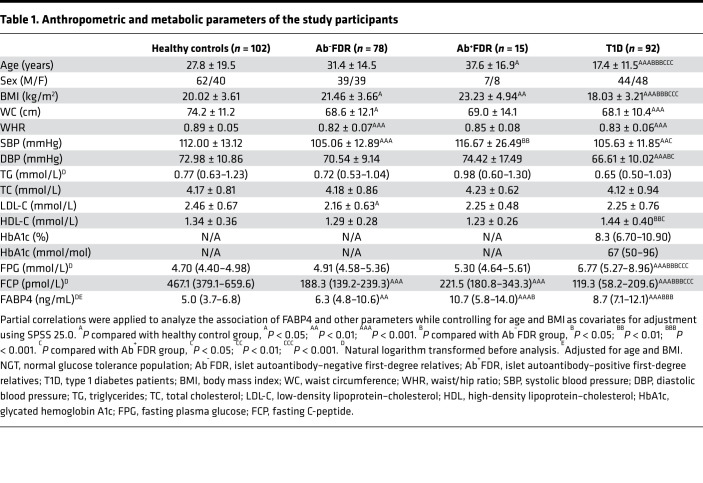
Anthropometric and metabolic parameters of the study participants

**Table 2:**
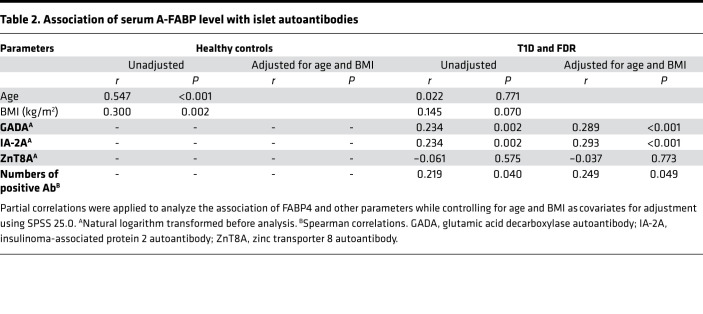
Association of serum A-FABP level with islet autoantibodies

## Version 1 03/09/2021

In-Press Preview

## Version 2 04/08/2021

Electronic publication
