# Supplementary material for: Fatty acid binding protein 4 promotes autoimmune diabetes by recruitment and activation of pancreatic islet macrophages
Source: JCI Insight. 2021 Apr 8;6(7):e141814. doi: 10.1172/jci.insight.141814 (PMC8119222; doi:10.1172/jci.insight.141814)
Supplement: Supplemental data [file jciinsight-6-141814-s128.pdf]

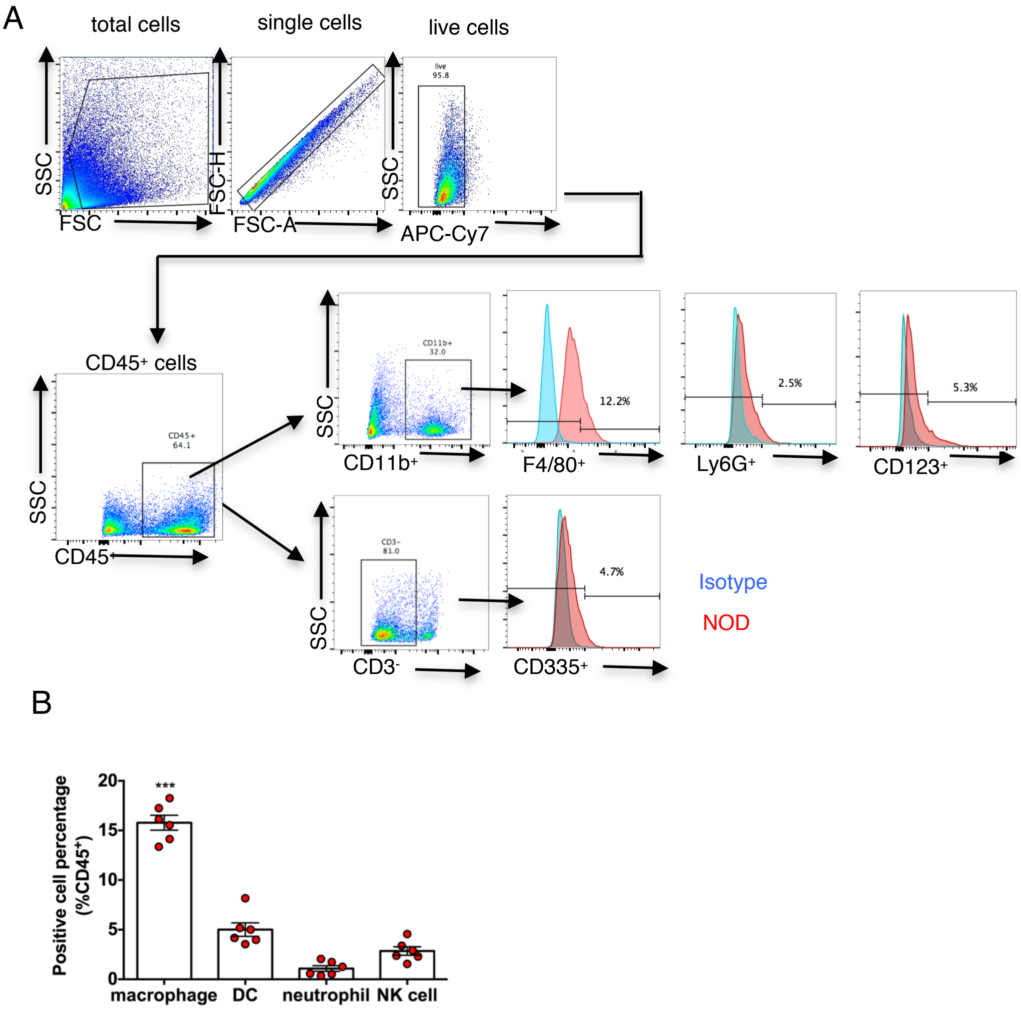

Supplementary Figure 1. Abundance of innate immune cells in pancreas of 6-week-old NOD/shiLtJ mice. (A) Representative FACS plots showing the abundance of macrophages ($CD45^+F4/80^+CD11b^+$), dendritic cells ($CD45^+CD123^+CD11b^+$), neutrophils ($CD45^+Ly6G^+CD11b^+$) and NK cells ($CD45^+CD3^-CD335^+$) among $CD45^+$cells in islets of 6-week-old NOD/shiLtJ mice, (n=6). **(B)** Quantification the percentage of macrophages ($F4/80^+CD11b^+$) and dendritic cells ($CD123^+CD11b^+$), neutrophils ($Ly6G^+CD11b^+$) and NK cells ($CD3^-CD335^+$) from live $CD45^+$ cells in islet of 6-week-old NOD/shiLtJ mice, (n=6). Data are expressed as mean ± standard deviation. ***$p<0.001$.

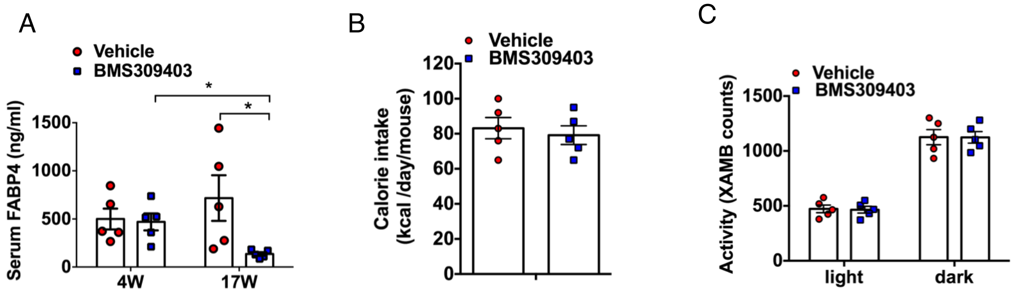

Supplementary Figure 2. Treatment with BMS309403 does not affect food intake and physical activity in mice. Female 4-week-old NOD mice were treated with BMS309403 (40 mg kg^{-1} d^{-1}) or vehicle by oral gavage for 8 weeks**. (A)** Circulating level of FABP4 in NOD mice treated with BMS309403 or vehicle at 4-week and 17-week old. **(B)** Calorie intake of NOD mice treated with BMS309403 (40 mg kg^{-1} d^{-1}) or vehicle. **(C)** Physical activity of above mice were determined by comprehensive laboratory animal monitoring system (CLAMS) analysis. Data are expressed as mean ± standard deviation. N=5.

A

B

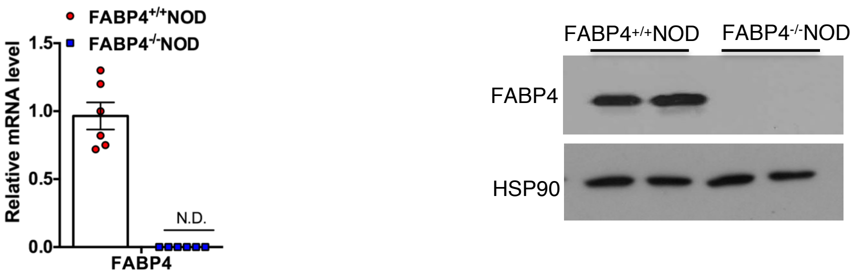

Supplementary Figure 3. Characterization of FABP4$^{+/+}$NOD and FABP4$^{-/-}$NOD mice. (A) The mRNA abundance of FABP4 in pancreas of FABP4$^{+/+}$NOD and FABP4$^{-/-}$NOD mice. **(B)** Representative immunoblots of FABP4 and HSP90 in pancreas isolated from FABP4$^{+/+}$NOD and FABP4$^{-/-}$NOD mice. Data are expressed as mean ± standard deviation. N=6.

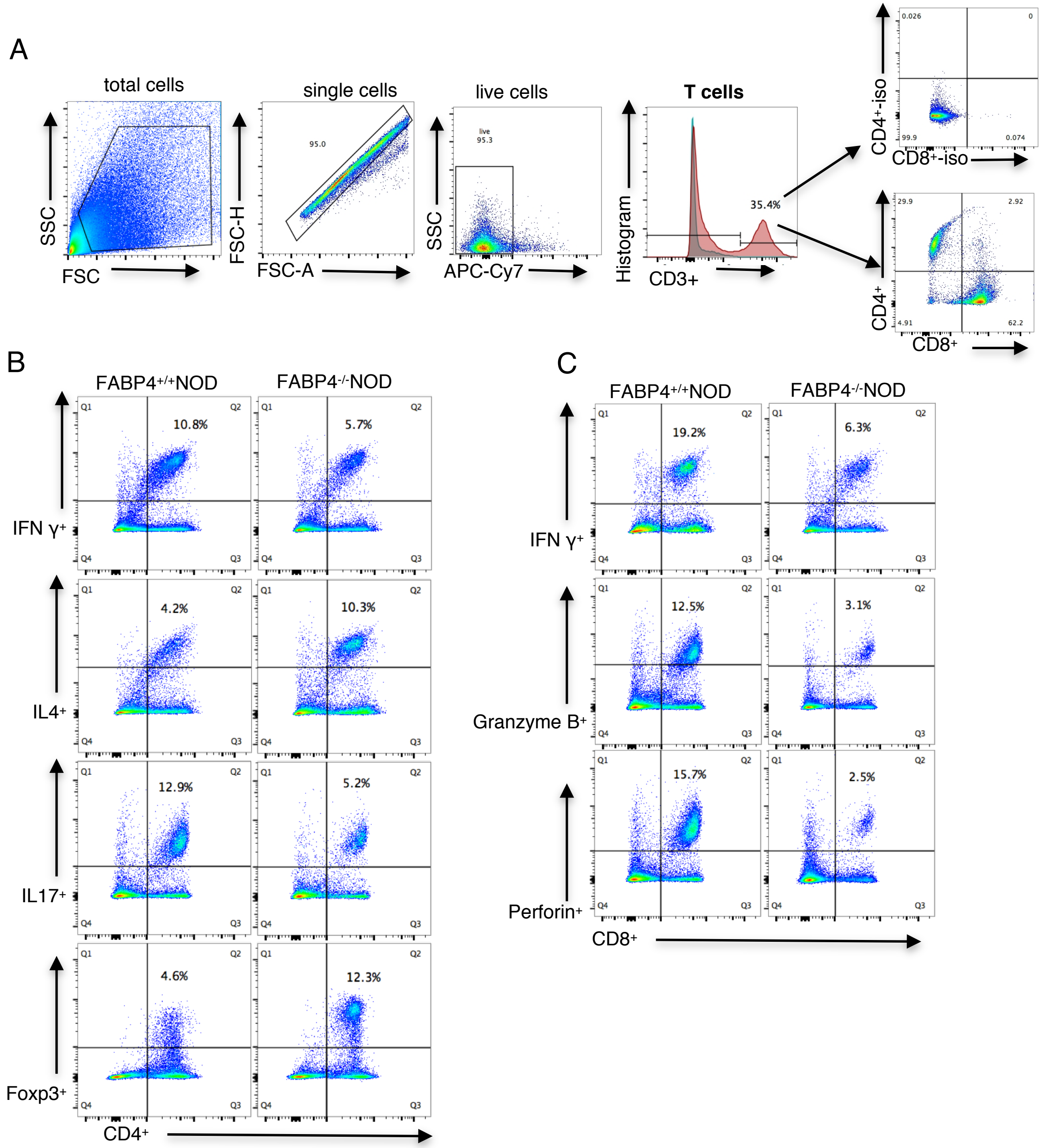

Supplementary Figure 4. FABP4 deficiency reduces diabetogenic T cells. (A) Gating strategy for Th ($CD3^+CD4+$) T cells and cytotoxic T (CD3+CD8+) T cells in islets of 10-week-old NOD mice. **(B)** Representative FACS plots showing the abundance of of Th1 ($IFN\gamma^+CD4^+$), Th2 ($IL4^+CD4^+$), Th17 ($IL17^+CD4^+$) and Treg ($Foxp3^+CD4^+$) cells in pancreas of 10-week-old $FABP4^{+/+}$NOD and $FABP4^{-/-}$NOD mice. **(C)** Representative FACS plots showing the abundance of IFNγ, granzymes B and perforin levels in cytotoxic T cell ($CD3^+CD8^+$) in pancreas of 10-week-old $FABP4^{+/+}$NOD and $FABP4^{-/-}$NOD mice. N=5.

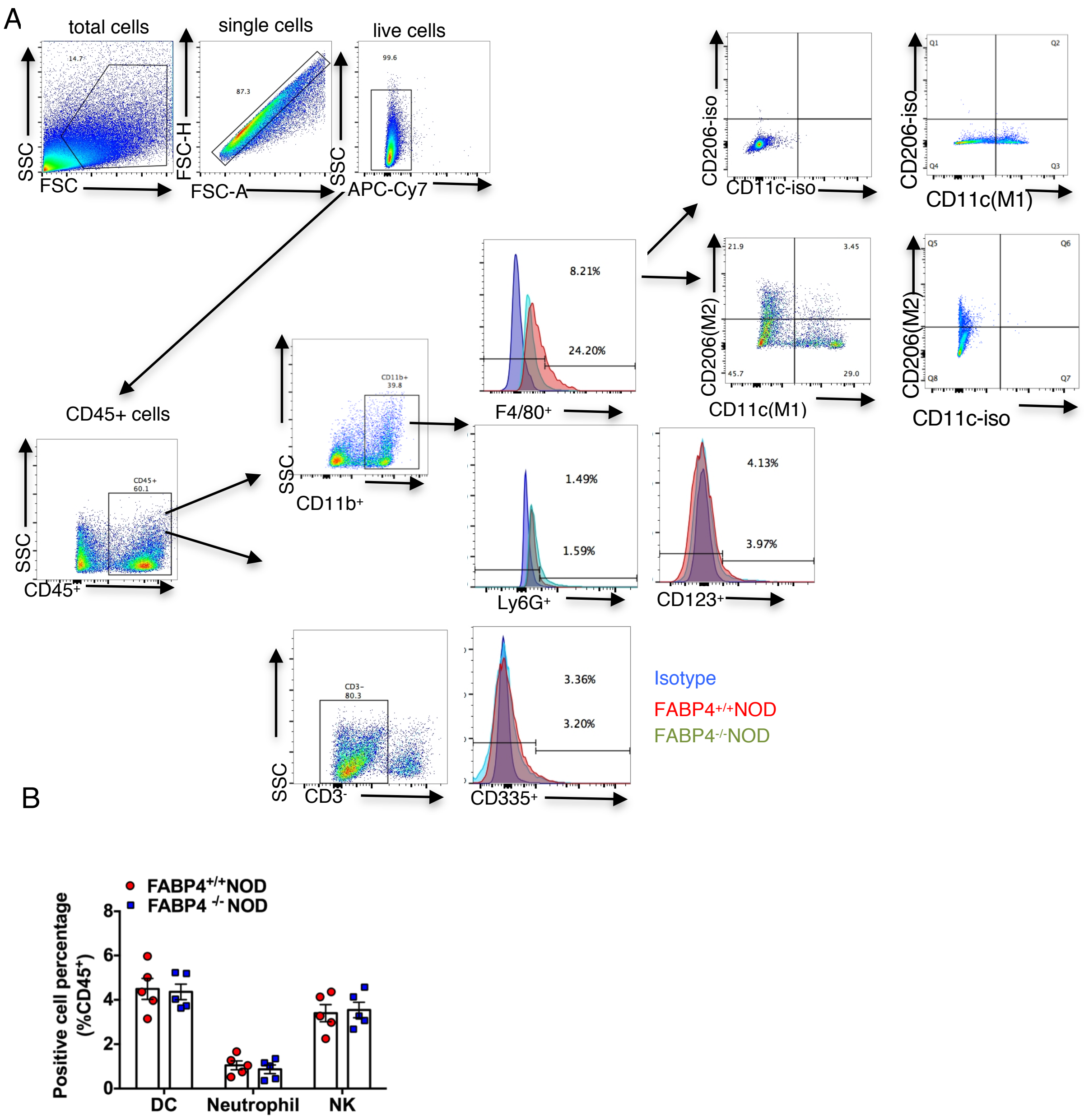

Supplementary Figure 5. FABP4 deficiency does not alter the infiltration of DCs, neutrophils and NK cells in pancreas of NOD mice. (A) Representative FACS plots showing the gating strategy and abundance of macrophages (CD45$^+$CD11b+F4/80) and their subtypes (M1:CD11c$^+$, M2:CD206$^+$), DCs (CD45$^+$CD11b$^+$CD123$^+$), neutrophils (CD45$^+$CD11b$^+$Ly6G$^+$) and NK cells (CD45$^+$CD3$^-$CD335$^+$), and **(B)** Quantification of mean frequency values of these cells in pancreas among CD45$^+$ cells of 6-week-old FABP4$^{+/+}$NOD and FABP4$^{-/-}$NOD mice. Data are expressed as mean ± standard deviation, *p<0.05, n=5.

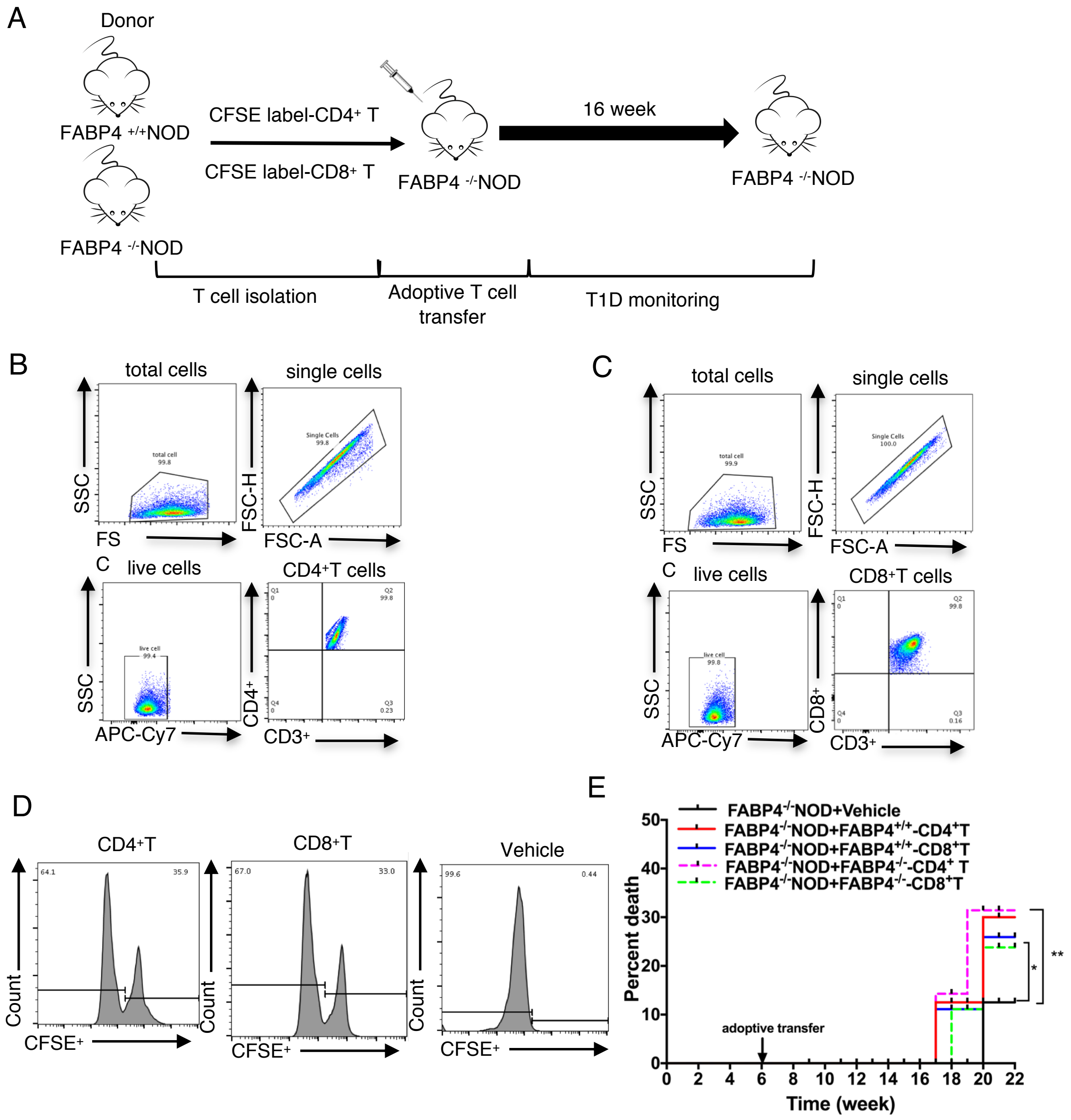

Supplementary Figure 6. FABP4 does not alter the diabetogenic effect of T cells in FABP4$^{-/-}$NOD mice. (A) Schematic diagram showing the experimental protocol of adoptive transfer of diabetogenic T cells into FABP4$^{-/-}$NOD mice. CD4^{+}T or CD8^{+}T cells (5×10^{6} cells) were isolated from 6-week-old FABP4$^{+/+}$NOD or FABP4$^{-/-}$NOD mice using magnetic beads (Miltenyi Biotech), labelled with 5 μM CFSE in PBS, and adaptively transferred into 6-week old FABP4$^{-/-}$NOD mice (i.p), followed by monitoring diabetes incidence for 16 weeks (n=15). **(B-C)** Representative flow cytometry confirming the purity of isolated of (**B**) CD4^{+} or **(C)** CD8^{+}T lymphocytes. **(D)** Confirmation of the success of adoptive transfer of CFSE-labelled CD4^{+}T or CD8^{+} T cells in the pancreatic lymph nodes of recipient FABP4$^{-/-}$NOD mice by flow cytometry analysis (n=5). **(E)** Diabetes incidence in FABP4$^{-/-}$NOD mice at different ages after adoptive transfer of diabetogenic T cells were compared using Pearson's chi-square test (n=10), *$p<0.05$.

Appendix Table

Sequences of primers for FABP4 genotype (Sequence 5'-3')	
FABP4 WT S2 (Forward)	CTTCTGAGGTGCACTCTATCCTC
FABP4 KO S1 (Forward)	TGCATCGCATTGTCTGAGTAGGTG
FABP4 CONAS1 (Reverse)	AGGTCTTGTATGCCACAGCGGAC

Supplementary table 1. Sequences of the primers used for genotyping in this study.

Gene name	5'-3' sequence	3'-5' sequence
Mouse FABP4	ACA CCG AGA TTT CCT TCA AAC TG	CCA TCT AGG GTT ATG ATG CTC TTC A
Mouse MCP-1	CCACTCACCTGCTGCTACTCA	TGGTGATCCTCTTGTAGCTCTC C
Mouse TNF α	CCCTCACACTCAGATCATCTTCT	GCTACGACGTGGGCTACAG
Mouse IL-1β	CTTCTTCGACACATGGGATAAC	TTTGGGATCTACACTCTCCAGC
Mouse IFN γ	AGA GGA TGG TTT GCA TCT GGG TCA	ACA ACG CTA TGC AGC TTG TTC GTG
Mouse ArgI	TGGCTTGCGAGACGTAGAC	GCTCAGGTGAATCGGCCTTTT
Mouse iNOS	CCAAGCCCTCACCTACTTCC	CTCTGAGGGCTGACACAAGG
Mouse GADPH	ACTCCCACTCTTCCACCTTC	TCTTGCTCAGTGTCCTTGC
Human FABP4	GGCATGGCCAAACCTAACAT	TTCCATCCCATTTCTGCACAT
Human GADPH	CGCCACAGTTTCCCGGAGGG	CCCTCCAAAATCAAGTGGGG

Supplementary table 2. Sequences of the primers used for real-time PCR in this study. Related to RNA Extraction and Real-Time PCR in STARMETHODS.

Antibodies	Source	Identifier
Rat anti-mouse F4/80 (clone BM8)	Biolegend	Cat#123107, RRID AB_893500
Hamster against mouse CD11c-PE (clone HL3)	BD Biosciences	Cat#553802, Entrez Gene ID 16411
Rat anti-mouse CD206-Alexa Fluor® 647 (clone C068C2)	Biolegend	Cat#141712, RRID AB_10900420
Rat anti-mouse CD11b-BV421 (Clone M1/70)	BD Biosciences	Cat#562605, Entrez Gene ID 16409
Rat anti-mouse CD123-PE (clone 5B11)	Biolegend	Cat#106005, RRID AB_2124403
Rat anti-mouse CD335-APC (clone 29A1.4)	Biolegend	Cat#137608, RRID AB_10612758
Rat anti-mouse Ly-6G-FITC (clone 1A8)	Biolegend	Cat#127605, RRID AB_1236488
rat anti-mouse CD3-APC (clone 17A2)	Biolegend	Cat#100236, RRID AB_2561456
rat anti-mouse CD4-FITC (clone GK1.5)	Biolegend	Cat#100406, RRID AB_312691
rat anti-mouse CD8a-PE (clone 53-6.7)	BD Biosciences	Cat#561095, RRID AB_312691
rat anti-mouse CD4-Alexa Fluor® 594 (clone GK1.5)	Biolegend	Cat#100446, RRID AB_2563182
rat anti-mouse CD8a-Alexa Fluor® 594 (clone 53-6.7)	Biolegend	Cat#100758, RRID AB_2563237
Rat anti-mouse Foxp3-PE (clone R16715)	BD Biosciences	Cat#563101
Rat anti-mouse IFN-γ-Alex Fluor 647 (clone XMG1.2)	BD Biosciences	Cat#557735
Rat anti-mouse IL-4-PE (clone 11B11)	BD Biosciences	Cat#554435
Rat anti-mouse IL-17A-PE (clone TC11-18H10)	BD Biosciences	Cat#559502
Mouse anti-human/mouse granzyme B-PE (clone QA16A02)	Biolegend	Cat#372208, RRID AB_2687032
rat anti-mouse perforin-PE (clone S16009B)	Biolegend	Cat#154406, RRIDAB_2721641
insulin	HyTest	Cat#2IP10-D6C4
Cleaved Caspase-3 (Asp175) (5A1E) Rabbit mAb	Cell Signaling	Cat#9664
Anti-F4/80 antibody [CI:A3-1]	Abcam	Cat#6640
Mouse/Rat FABP4/A-FABP Antibody	R&D Systems	Cat#AF1443
HSP90 (C45G5) Rabbit mAb	Cell Signaling	Cat#4877

Continued

Chemicals, Peptides, and Recombinant Proteins	Source	Identifier
Red blood cell (RBC) lysis buffer	BioLegend	Cat#420301
LIVE/DEAD fixable near-IR dead cell stain	Molecular Probes	Cat#L34975
Fixation/Permeabilization kit	eBioscience	Cat#00-5523
Protease inhibitor cocktail tablets	Roche	Cat#04 693 116 001
Enhanced chemiluminescence reagents	GE Healthcare	Cat#RPN2232
RNAiso Plus	Takara	Cat#9109
PrimeScript RT reagent kit	Takara	Cat#RR037A
SYBR Premix Ex Taq	Takara	Cat#RR420D
Gadolinium trichloride (Gdcl3)	Sigma–Aldrich	Cat#439770
Collagenase P	Roche Diagnostics	Cat#11213857001
phorbol 12-myristate 13-acetate (PMA）	Sigma–Aldrich	Cat#P8139
ionomycin	Sigma–Aldrich	Cat#Calbiochem407953
GolgiStopTM protein transport inhibitor	BD Biosciences	Cat#554724

Critical Commercial Assays	Source	Identifier
Adipocyte FABP (FABP4) Mouse ELISA	Biovendor	Cat# RD291036200R
Mouse Insulin ELISA, Ultra sensitive	Immunodiagnostics	Cat# 32380

Oligonucleotides	Source	Identifier
Real-time PCR primers used in this study	See Table S1	N/A
Primers used for genotyping in this study	See Table S2	N/A

Continued		
**Experimental Models: Organisms/Strains**	**Source**	**Identifier**
NOD/ShiLtJ	The Jackson Laboratory	Stock No: 001976
FABP4$^{-/-}$ in C57BL/6N background	Shu et al. 2017.	N/A
**Software and instrument**	**Source**	**Identifier**
FlowJo version X.0.7	Tree Star	https://www.flowjo.com/solutions/flowjo
Image J	National Institutes of Health (NIH)	https://imagej.nih.gov/ij/
GraphPad Prism 7	GraphPad	https://www.graphpad.com/

Supplementary table 3. Key resources table.
